# Multimodal chromatin profiling using nanobody-based single-cell CUT&Tag

**DOI:** 10.1038/s41587-022-01535-4

**Published:** 2022-12-19

**Authors:** Marek Bartosovic, Gonçalo Castelo-Branco

**Affiliations:** 1 Laboratory of Molecular Neurobiology, Department of Medical Biochemistry and Biophysics, Karolinska Institutet, Stockholm, Sweden; 2 Ming Wai Lau Centre for Reparative Medicine, Stockholm node, Karolinska Institutet, Stockholm, Sweden; 3 Present Address: Department of Biochemistry and Biophysics, Stockholm University, Stockholm, Sweden

**Keywords:** Chromatin analysis, Epigenomics, Cellular neuroscience, Histone post-translational modifications

## Abstract

Probing histone modifications at a single-cell level in thousands of cells has been enabled by technologies such as single-cell CUT&Tag. Here we describe nano-CUT&Tag (nano-CT), which allows simultaneous mapping of up to three epigenomic modalities at single-cell resolution using nanobody-Tn5 fusion proteins. Multimodal nano-CT is compatible with starting materials as low as 25,000–200,000 cells and has significantly higher sensitivity and number of fragments per cell than single-cell CUT&Tag. We use nano-CT to simultaneously profile chromatin accessibility, H3K27ac, and H3K27me3 in juvenile mouse brain, allowing for discrimination of more cell types and states than unimodal single-cell CUT&Tag. We also infer chromatin velocity between assay for transposase-accessible chromatin (ATAC) and H3K27ac in the oligodendrocyte lineage and deconvolute H3K27me3 repressive states, finding two sequential waves of H3K27me3 repression at distinct gene modules during oligodendrocyte lineage progression. Given its high resolution, versatility, and multimodal features, nano-CT allows unique insights in epigenetic landscapes in complex biological systems at the single-cell level.

## Abstract (web summary)

Three epigenetic features are mapped at single-cell resolution using Tn5-nanobody fusion proteins

## Main

Cell identity and the underlying gene expression programs are determined through the action of epigenetic modalities including transcription factor binding^1^, modifications of histones^2^, chromatin remodeling^3^, DNA methylation^4^, genome architecture^5^ and long non-coding RNAs^6^. Together, effects of these factors determine the regulatory logic behind cell state transitions during development and in disease. Changes in the chromatin state, for example chromatin opening^7,8^, priming^9^, or enhancer RNA expression^10^ may precede gene transcription. Therefore, comprehensive and multimodal chromatin maps could be used to predict cell lineage or state commitment before the transcriptional program has been activated.

The chromatin regulatory principles include co-binding of multiple epigenetic factors such as transcription factors, and co-occurrence of synergistic or antagonistic histone marks. For example, *cis*-regulatory elements are often characterized by co-occurrence of several active histone marks^11^ and in bivalent loci, active and repressive histone marks coincide^12^. These epistatic interactions between epigenetic modalities are considered important for rapid activation and repression of gene expression but they are still not yet fully understood.

Novel single-cell resolution, epigenome profiling technologies such as single-cell assay for transposase-accessible chromatin with sequencing (scATAC-seq), single-cell CUT&Tag (scCUT&Tag) have used Tn5 transposase or its fusion proteins and have provided unprecedented resolution in chromatin profiling^13–24^. These technologies enabled the detection of changes in the chromatin states in samples with unknown heterogeneity and in dynamically changing systems, such as during development and differentiation. Furthermore, integration of single-cell epigenomics with other -omics datasets have provided additional insights into the interplay between epigenetics and transcription^25,26^. However, single-cell -omics data integration relies on correlation of features across modalities, which is limiting, whereas direct measurement of multiple modalities in the same cell can bring more meaningful insights into chromatin functions.

Currently, multimodal profiling of chromatin and gene expression have made it possible to directly link changes in the epigenome with gene expression changes at single-cell resolution^8,23,27^. These methods provided critical insights into changes in chromatin that precede gene activation, transcription, and expression. Recently, technologies that profile two epigenetic modalities in single cells have been developed^28,29^. Among these, multi-CUT&Tag uses proteinA–Tn5 fusion pre-complexed with barcoded oligonucleotides^28^. Multi-CUT&Tag can profile various combinations of epigenetic modalities but has limited sensitivity—low number of unique fragments per cell and has only been demonstrated to work at a single-cell level in a mixture of cell lines. Low sensitivity might make it difficult to deconvolute subtle epigenetic differences between related cell types and cell states within a complex tissue.

Another technology—scGET-seq—implemented a novel fusion of Tn5 to the H3K9me3 reader HP1α complexed with barcoded oligonucleotides to profile heterochromatin, and barcoded ATAC-seq to profile open chromatin at the same time in single cells^29^. Although scGET-seq provides higher sensitivity than multi-CUT&Tag, it lacks the flexibility of CUT&Tag to profile various modalities. Even though scGET-seq could in principle be used together with pA-Tn5 profiling, two out of three modalities (HP1α and ATAC-seq) would still be invariable.

Here we describe nanobody-based scCUT&Tag (nano-CT), which can be used to profile three epigenetic modalities simultaneously in single cells using Tn5 fusion proteins coupled with secondary nanobodies^30^. The nanobodies target either mouse or rabbit primary antibody and thus profile any combination of two histone modifications at the same time. We also demonstrate that nano-CT can be combined with scATAC-seq to profile open chromatin simultaneously, altogether profiling three epigenetic modalities. Furthermore, we improved the CUT&Tag tagmentation and library preparation protocol so that nano-CT requires less input material and provides higher depth per cell than previous scCUT&Tag iterations^14–16,31^. The modular design of the nano-Tn5 makes it possible to profile a variety of different combinations of chromatin modalities and nano-CT will undoubtedly be an important addition to the spectrum of emerging multimodal single-cell epigenome profiling technologies.

## Results

### Unimodal single-cell nano-CT outperforms scCUT&Tag

**Fig. 1:**
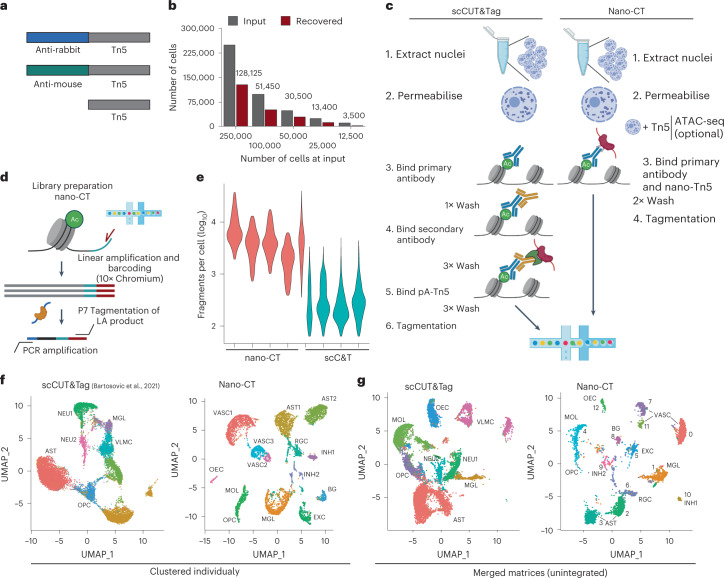
nano-CUT&Tag (nano-CT). **a**, Schematic image of the Tn5 fusion proteins used in the experiments. **b**, Bar plot depicting number of cells used as input for nano-CT and number of cells recovered. **c**, Comparison of the antibody- and Tn5-binding strategy between scCUT&Tag and nano-CT. **d**, Cartoon depiction of the tagmentation and library preparation strategy. The nano-Tn5 is loaded with MeA/Me-Rev oligonucleotides, tagmented genomic DNA is used as template for linear amplification, which is then tagmented in a second round with standard Tn5 loaded with MeB/Me-Rev oligonucleotides. The resulting library is amplified by PCR and sequenced. **e**, Violin plot depicting number of unique reads per cell obtained by scCUT&Tag^15^ and nano-CT targeting H3K27me3 per replicate. Violin plots 1–4 from left show multimodal nano-CT performed without ATAC (1 and 3 from left) or with ATAC-seq (2 and 4 from left), and violin plot 5 depicts unimodal nano-CT experiment. **f**, Individual UMAP embeddings of the single-modality scCUT&Tag (left) and nano-CT (right) data depicting the identified clusters, (scCUT&Tag: 13,932 cells in 4 biological replicates; nano-CT: 6,798 cells in 1 biological replicate; 200,000 cells used as input) **g**, UMAP co-embedding of the scCUT&Tag data (13,932 cells in 4 biological replicates) together with nano-CT data (6,798 cells in 1 biological replicate; 200,000 cells used as input). Raw matrices obtained by scCUT&Tag and nano-CT were merged together and analyzed without integration. VASC, vascular; AST, astrocytes; RGCs, radial glial cells; OECs, olfactory ensheathing cells; OPCs, oligodendrocyte progenitor cells; MOLs, mature oligodendrocytes; BG, bergman glia; EXC, excitatory neurons; INH, inhibitory neurons; MGL, microglia.

To multiplex the profiling of chromatin states in the same single cell, we designed a single-chain secondary antibody^30^—nanobody-Tn5 fusion proteins (nano-Tn5; Fig. 1a), conferring the specificity of the Tn5 towards primary antibodies raised in either mouse (ms-Tn5) or rabbit (rb-Tn5). Because nano-Tn5 binds directly to the primary antibodies, secondary antibody incubation from the CUT&Tag protocol is not required and is thus omitted (Fig. 1c). The monovalent interaction of the nanobody with the primary antibody further allows to combine the antibody and nano-Tn5 incubation steps, greatly simplifying the nano-CT protocol and reducing the number of required washes and increasing the yield of recovered nuclei ranging between 28 to 68% (Methods; Figs. 1b,c). This allows profiling of low input bulk samples from as little as ~25,000 cells as starting material, compared to previously required 1 million^16,32^ or 150,000^15^ cells for scCUT&Tag protocols on the Chromium platform.

**Extended Data Fig. 1:**
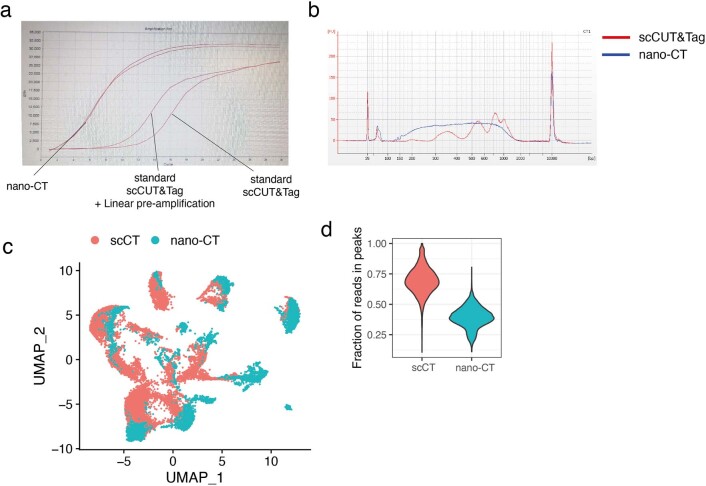
Comparison of nano-CUT&Tag and scCUT&Tag H3K27me3 profiles. **a**. qPCR amplification curve for libraries generated using the same amount of input material using scCUT&Tag method, scCUT&Tag combined with linear pre-amplification and nano-CT protocol with two-step tagmentation. **b**. Bioanalyser plot showing the size distribution of libraries generated using scCUT&Tag and nano-CT. Typical nucleosome profile can only be seen in the scCUT&Tag library. **c**. UMAP co-embedding of the scCUT&Tag and nano-CT dataset (single modality). The raw count matrices were merged, and dimensionality reduction was done on both datasets together. **d**. Violin plot showing fraction of reads in peak regions (FrIP) for data collected by scCUT&Tag and nano-CT.

We further developed a tagmentation and nano-CT library prep protocol for single-cell applications (Fig. 1d). We first perform a deterministic tagmentation to capture more sites using a nano-Tn5 loaded with MeA/Me-Rev (P5) oligonucleotides. The tagmented genomic DNA is then used as a template for combined linear amplification and barcoding on the 10x Genomics Chromium platform. After the recovery of the barcoded DNA, a second adapter is introduced using second tagmentation with MeB/Me-Rev (P7)-loaded standard Tn5, followed by library amplification by PCR (Fig. 1d). This tagmentation protocol led to decreased cycle requirements for PCR amplification of the library in a bulk experiment, from 15 cycles to around 6 cycles (Extended Data Fig. 1a) yielding a library with similar concentration. The nucleosome phasing size profile typical for scCUT&Tag is not present in the nano-CT library (Extended Data Fig. 1b). The reduction in the number of PCR cycles reflects higher capture efficiency by the nano-Tn5 fusion protein and tagmentation protocol when compared to conventional CUT&Tag and can’t be explained solely by the introduction of linear pre-amplification step (Extended Data Fig. 1a).

We first tested the newly developed protocol by profiling of H3K27me3 in post-natal day 19 (P19) mouse brain. We obtained 6,798 single-cell profiles with a median of 3,720 unique fragments, which is 15.8-fold increase in the number of unique fragments per cell over the previous generation of scCUT&Tag technology when performed on the same platform and tissue (Fig. 1e). The increase in the number of unique fragments was associated with a reduction in the fraction of reads in peak regions by about 1.7-fold from 69% to 39% when using the same peak-calling parameters (Extended Data Fig. 1d).

We then constructed a 5-kilobase-bin-by-cell matrix, performed clustering and dimensionality reduction using latent semantic indexing (LSI) and uniform manifold approximation and projection (UMAP) and identified 13 clusters (Fig. 1f). We merged the newly generated data with the previous generation of scCUT&Tag data^15^ by combining the raw count matrices and performed dimensionality reduction (Fig. 1g). The individual clusters intermingled well among the technologies (Extended Data Fig. 1c) without using integration methods underscoring the reproducibility of the nano-CT protocol with previous scCUT&Tag technology (Fig. 1g).

**Extended Data Fig. 2:**
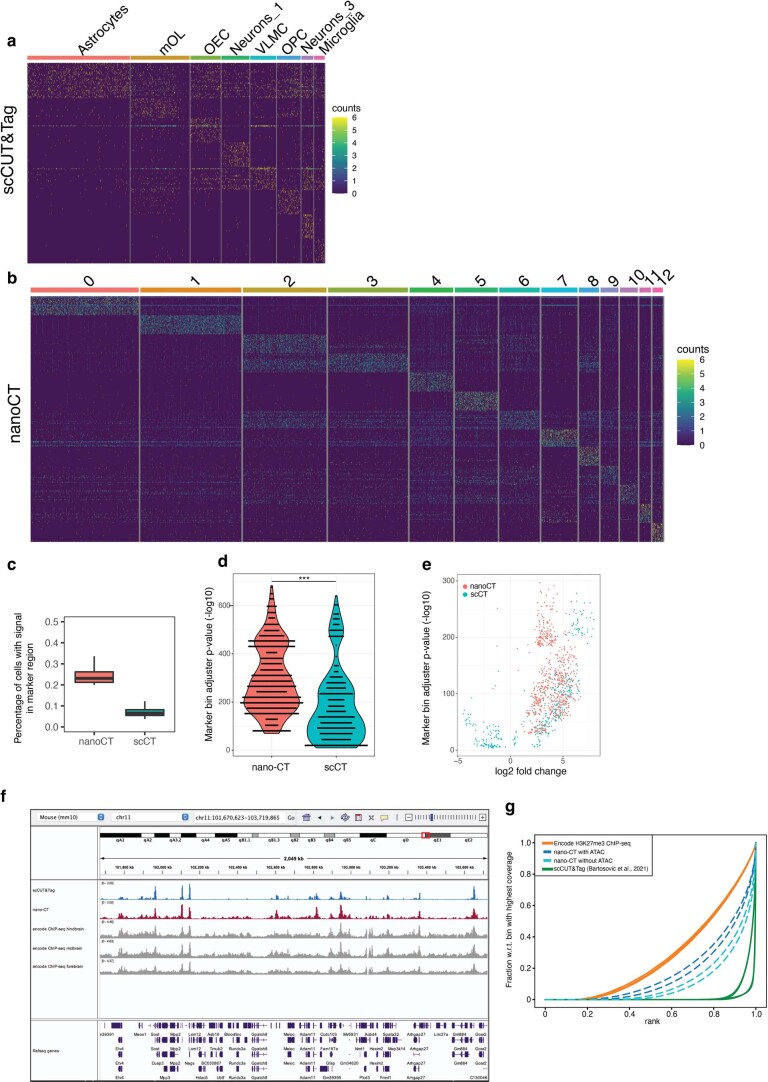
Benchmarking and QC of marker regions identified by nano-CUT&Tag. **a**. Heatmap showing the cell by marker matrix for scCUT&Tag dataset. Top markers were selected based on adjusted p-value calculated using Seurat’s FindAllMarkers function (two-sided Wilcoxon rank sum test and corrected for multiple hypothesis testing by Bonferroni correction). **b**. Heatmap showing the cell by marker matrix for nano-CT dataset. Top markers were selected based on adjusted p-value calculated using Seurat’s FindAllMarkers function (two-sided Wilcoxon rank sum test and corrected for multiple hypothesis testing by Bonferroni correction). **c**. Boxplot depicting fraction of cells with any signal (counts >1) in the most significantly enriched marker regions (top 100) for the respective cluster. Markers are selected based on adjusted p-value calculated using Seurat’s FindAllMarkers function (two-sided Wilcoxon rank sum test and corrected for multiple hypothesis testing by Bonferroni correction). Lower and upper bounds of boxplot specify 25th and 75th percentile, respectively, and lower and upper whiskers specify minimum and maximum, respectively, no further than 1.5× interquartile range. Outliers are not displayed. **d**. Violin jitter plot showing the p-value determined by two-sided Wilcoxon rank sum test (Seurat FindAllMarkers function), distribution for the top 50 marker bins for each cluster of nano-CT and scCUT&Tag dataset. *** p value = 1.3×10^−34^ (Two sided wilcoxon rank sum test) **e**. Volcano plot showing p-value determined by two sided wilcoxon rank sum test (Seurat FindAllMarkers function) and average log2 fold change for top 50 marker bins in nano-CT and scCUT&Tag. **f**. Genome browser snapshot showing pseudobulk scCUT&Tag, nano-CT and Encode dataset (forebrain, midbrain and hindbrain) for H3K27me3 histone mark. **g**. Fingerprint analysis of H3K27me3 pseudobulk scCUT&Tag, nano-CT and encode datasets.

We could identify and deconvolute more fine-grained clusters from the nano-CT data compared to the scCUT&Tag. For example, we deconvoluted the cluster containing astroependymal cells into four new sub-clusters (clusters 2, 3, 6, and 8) and the cluster vascular lepto-meningeal cells (VLMCs) into three new sub-clusters (cluster 0, 7, and 11) (Fig. 1f,g). On the basis of H3K27me3 profiles, we also detected cells from the broad spectrum of oligodendrocyte lineage, in contrast to scCUT&Tag, which could deconvolute these intermediate cell states only upon integration with single-cell RNA sequencing (scRNA-seq) data (Fig. 1f). Moreover, the marker bins identified by single-cell nano-CT showed a significantly higher capture rate (23.1%) versus markers identified by scCUT&Tag (6.6%) (Extended Data Fig. 2a–c) and the top enriched markers (50 for each cluster) showed significantly higher *P* value (Extended Data Fig. 2d,e; Wilcoxon test). Although nano-CT data also showed increased levels of background noise comparing to scCUT&Tag data, the levels of background were below the background of benchmarking encode H3K27me3 ChIP-seq data^33^ in nano-CT datasets (Extended Data Fig. 2f,g).

### Multimodal single-cell nano-CT in the mouse brain

**Fig. 2:**
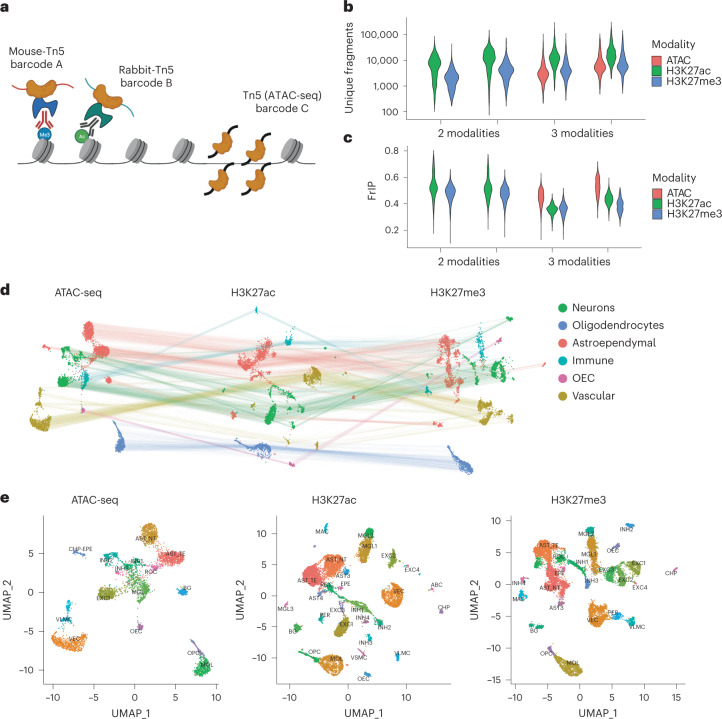
Multimodal nano-CT. **a**, Cartoon depicting the strategy used to profile multiple epigenomic modalities. Individual Tn5 and nano-Tn5 are loaded with barcoded oligonucleotides that are used in the analysis to identify the source of tagmentation and demultiplex the modalities. **b**, Violin plots depicting the number of unique fragments per cell per replicate and modality. **c**, Violin plots depicting FrIP per cell per replicate and modality. **d**, UMAP embeddings of the multimodal nano-CT data for ATAC-seq, H3K27ac, and H3K27me3. The lines connect representations of the same cells in the individual modalities (4,434 cells in two biological replicates, which passed quality control for all three modalities individually and originate from the three-modal datasets; 200,000 cells used as input for all replicates). **e**, UMAP embedding of the individual modalities with cluster labels. *n* = 2 biological replicates—each biological replicate was profiled both by nano-CT with ATAC (3-modal) and nano-CT without ATAC (2-modal): 4,960 cells ATAC-seq, 12,464 cells H3K27ac, 12,763 cells H3K27me3; 200,000 cells were used as input for all replicates. Cell is shown in modality UMAP if it passes quality control in its respective modality regardless of the other modalities. AST_NT, astrocytes non-telencephalon; AST-TE, astrocytes telencephalon; AST_3, astrocytes 3; AST_4, astrocytes 4; INH1–4, inhibitory neurons; EXC1–4, excitatory neurons; MGL1–3, microglia 1–3; MAC, macrophages; VEC, vascular endothelial cells; PER, pericytes; CHP, choroid plexus epithelial cells; EPE, ependymal cells; CHP-EPE, choroid plexus + ependymal cells; BG, Bergmann glia; VSMC, vascular smooth muscle cells; ABC, arachnoid barrier cells.

Next, we loaded the nanobody-Tn5 fusion proteins with different barcoded oligonucleotides to be able to track the insertions by distinct Tn5 fusion proteins (Fig. 2a and Supplementary Table 1). We performed nano-CT with single-cell indexing on the 10x Genomics Chromium ATAC-seq v1.1 platform on fresh mouse brain tissue obtained from 19-day-old mice (P19). By using uniquely barcoded oligonucleotides, we targeted two histone modifications simultaneously, H3K27ac and H3K27me3 (two biological replicates). In addition, we also profiled chromatin accessibility by a prior treatment with non-fused, barcoded Tn5 (ATAC-seq) in the same sample in two biological replicates, altogether profiling three epigenomic modalities in single cells (Fig. 2a). Performing ATAC-seq before nano-CT consistently resulted in increased nuclei loss and clumping and thus profiling of the three modalities required at least 200,000 cells as input material.

**Extended Data Fig. 3:**
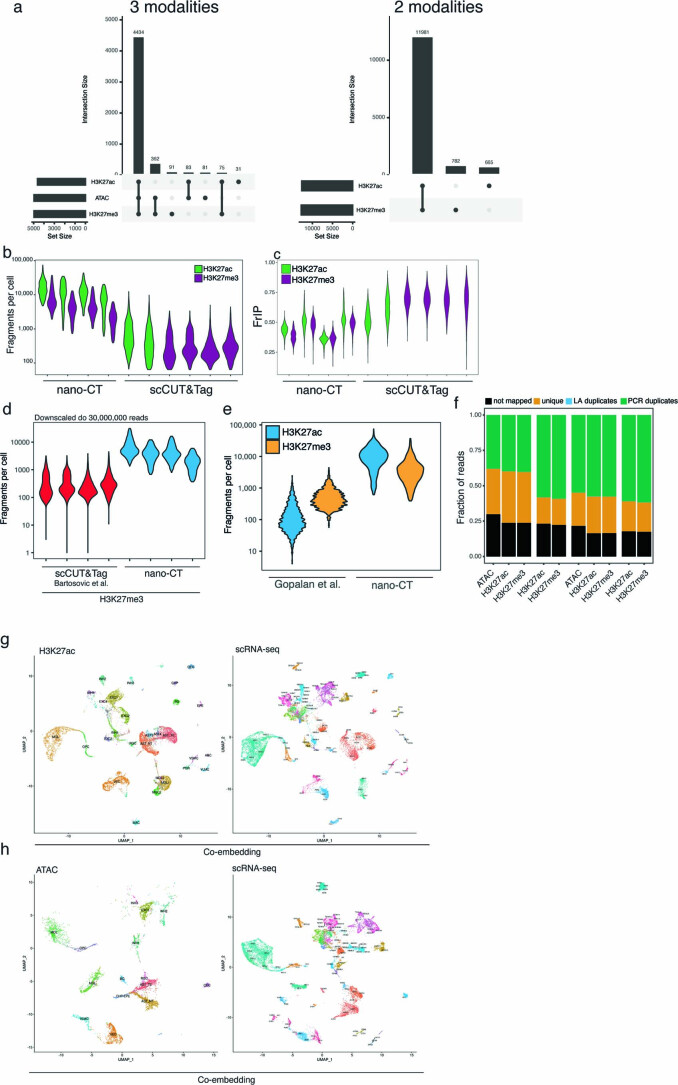
QC and integration of multimodal nano-CUT&Tag. **a**. Upset plots showing the overlap between cells that pass QC within the different modalities for combinations of H3K27ac and H3K27me3, and also ATAC, H3K27ac and H3K27me3. **b**. Violin plot showing the distribution of fraction of reads per cell in nano-CT and scCUT&Tag. **c**. Violin plot showing the distribution of number of reads per in peak regions (FrIP) in nano-CT and scCUT&Tag. **d**. Violin plot showing the number of fragments per cell for scCUT&Tag and nano-CT datasets downscaled to 30,000,000 reads for each dataset. **e**. Violin plot depicting the number of fragments per cell for multi-CUT&Tag^28^ and nano-CT and for two modalities (H3K27ac and H3K27me3). **f**. Fraction of reads not mapped, unique, result of linear amplification duplicates or result of PCR duplicates in nano-CT and multimodal nano-CT experiments. **g**. UMAP co-embedding of CCA-integrated H3K27ac nano-CT together with scRNA-seq dataset. Gene body and promoters were used as gene activity scores for the integration. **h**. UMAP co-embedding of CCA-integrated nano-CT ATAC-seq together with scRNA-seq dataset. Gene body and promoters were used as gene activity scores for the integration.

We demultiplexed the three modalities and preprocessed the datasets individually using the Cellranger pipeline (10x Genomics). We selected the cells using two parameters—number of reads per cell and fraction of reads in peak per cell—and identified the high-quality cells using Gaussian mixture model clustering in each modality separately (Supplementary Fig. 1). Out of 13,428 and 5,157 cells identified in the two-modality and three-modality datasets 11,981 (89.2%) and 4,434 (85.9%) cells passed the quality control filter in all modalities, respectively (Extended Data Fig. 3a). We then compared the number of unique fragments per cell and the fraction of reads in peaks (FrIP) with our previous scCUT&Tag dataset^15^. We found that multimodal nano-CT consistently outperforms previous scCUT&Tag protocol in terms of the number of fragments per cell in each modality (median 6,123–1,4496 and 1,832–6,510 fragments per cell for nano-CT and 315–547 and 217–329 fragments per cell for scCUT&Tag; H3K27ac and H3K27me3, respectively; Fig. 2b and Extended Data Fig. 3b), also after downscaling the individual replicates to the same sequencing depth (30 million reads per sample, median 2,949 reads per cell nano-CT, 221 scCUT&Tag in H3K27me3; Extended Data Fig. 3d). We also detected lower signal-to-noise ratio measured as the fraction of reads in peak regions (35.9–52.4% and 36.3–48.9% nano-CT and 51.2–62.1% and 67.9–69.9 scCUT&Tag; H3K27ac and H3K27me3, respectively; Fig. 2c and Extended Data Fig. 3c). Increase in number of profiled modalities also resulted in modest increases in assay noise as measured by fraction of reads in peak regions (Fig. 2c) and fingerprint analysis (Extended Data Fig. 2g). The change in the library construction strategy resulted in a minimal number of duplicate linear amplification fragments across all samples (0.11–0.14% of total reads; Extended Data Fig. 3f).

Nano-CT also vastly outperforms previously reported multimodal histone profiling method-multi-CUT&Tag in terms of number of fragments per cell with median 95 and 428 reads per cell in multi-CUT&Tag and 9,135 and 3,201 median reads per cell in nano-CT in H3K27ac and H3K27me3, respectively (Extended Data Fig. 3e).

**Fig. 3:**
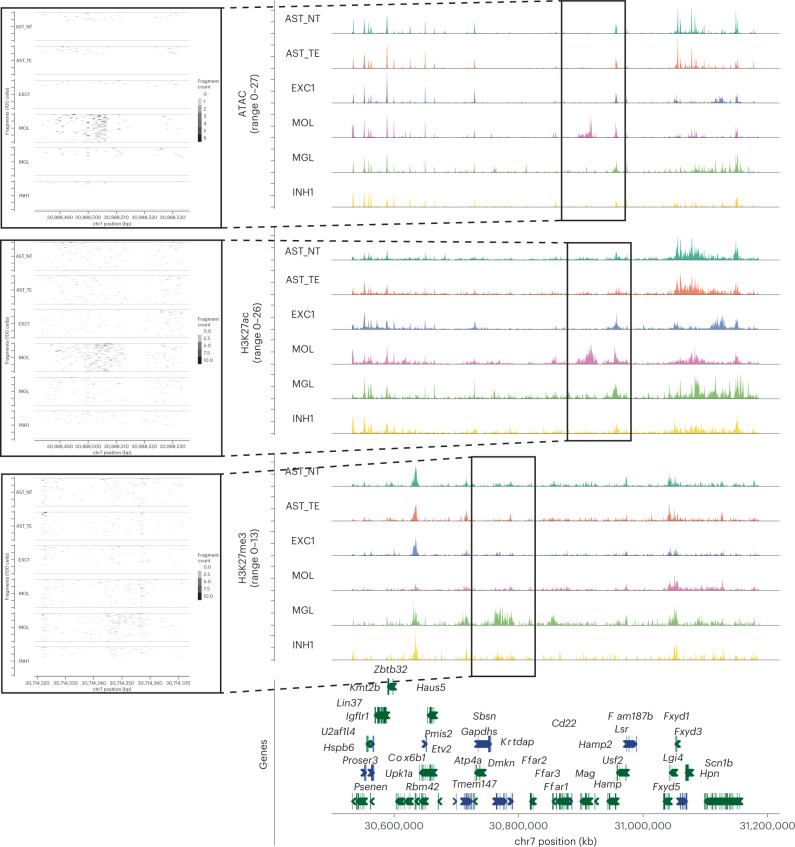
ATAC, H3K27ac and H3K27me3 at loci harboring microglia and mature oligodendrocyte marker genes. Genome browser tracks of the multimodal data for several clusters showing marker peak regions. Markers: *Mag* for ATAC/H3K27ac in mature oligodendrocytes and *Dmkn* for H3K27me3 in microglia.

**Extended Data Fig. 4:**
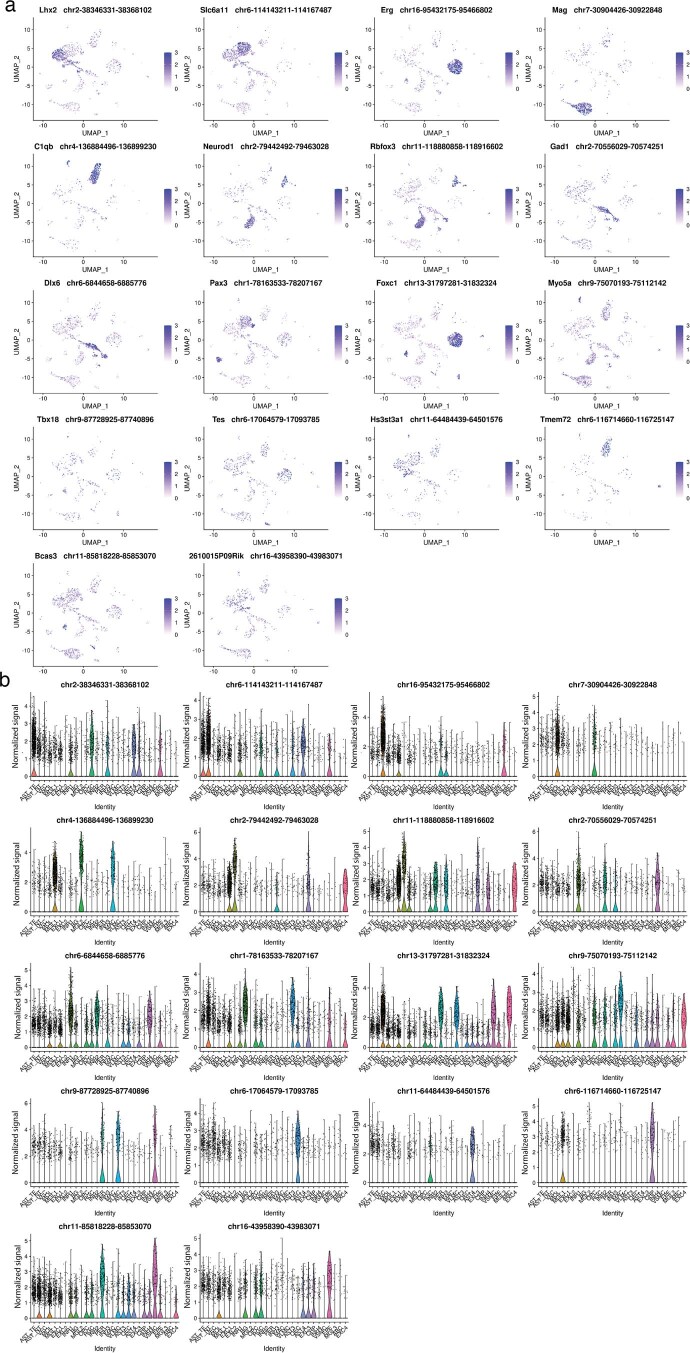
UMAP and violin plot visualization of marker H3K27ac regions. **a**. UMAP embedding and visualization of marker peak activities for H3K27ac nano-CT. Exact genomic region together with the closest gene is shown in the plot title. **b**. Violin plot visualization the of peak scores for the same peak regions as in a).

We then constructed cell-by-peak matrix for all modalities and performed dimensionality reduction using LSI, UMAP, and clustered the cells using each modality separately. We identified individual clusters in each modality, obtaining 15 ATAC-seq clusters, 28 H3K27ac clusters, and 24 H3K27me3 clusters and broadly classified them into major cell classes—neurons, oligodendrocytes, astroependymal, immune, and vascular cells (Fig. 2d,e). We then annotated the clusters by co-embedding the active modalities (ATAC and H3K27ac) together with the single-cell RNA-seq (scRNA-seq) mouse brain atlas dataset^34^ using canonical correlation analysis (CCA) (Extended Data Figs. 3g,h). All clusters displayed a combination of unique marker regions in all modalities including enhancers labeled by H3K27ac proximal to *Mag*/*Mbp* (OLG), *Rbfox3* (pan-NEU), *C1qa*/*C1qb* (microglia/macrophage), *Gad1* (inhibitory neurons), *Neurod1* (excitatory neurons), *Foxg1*/*Lhx2* (astrocytes telencephalon), *Irx2* (astrocytes non-telencephalon), and *Foxc1* (vascular endothelial cells/VLMCs/pericytes) among others (Fig. 3, Extended Data Figs. 4a,b).

**Fig. 4:**
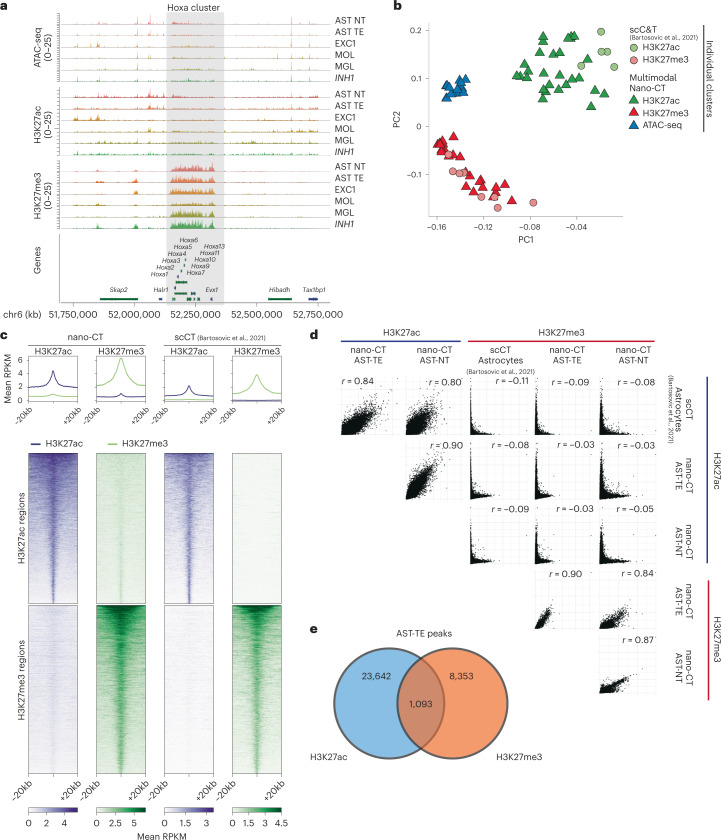
Quality control and benchmarking of nano-CT. **a**, Genome browser nano-CT pseudo-bulk view of the HoxA region on chromosome 6 for all three modalities. **b**, Principal component analysis (PCA) of pseudo-bulk tracks for each cluster identified from the respective modalities by scCUT&Tag and nano-CT. Top 50 marker regions were selected from each nano-CT cluster and modality, and all peaks were merged and flattened before running PCA. **c**, Metagene plots showing the signal distribution of H3K27ac and H3K27me3 in astrocyte populations obtained by nano-CT and scCUT&Tag around specific H3K27ac and H3K27me3 peaks. The peaks were defined and selected on the basis of reference scCUT&Tag data^15^. **d**, Scatter plots matrix showing correlation of H3K27ac and H3K27me3 signal in astrocyte populations defined by scCUT&Tag and nano-CT. *r*, Pearson correlation coefficient. Cell labels as in Fig. 2. **e**, Venn diagram showing the genomic overlap of significant H3K27ac and H3K27me3 peaks in cluster AST-TE.

**Extended Data Fig. 5:**
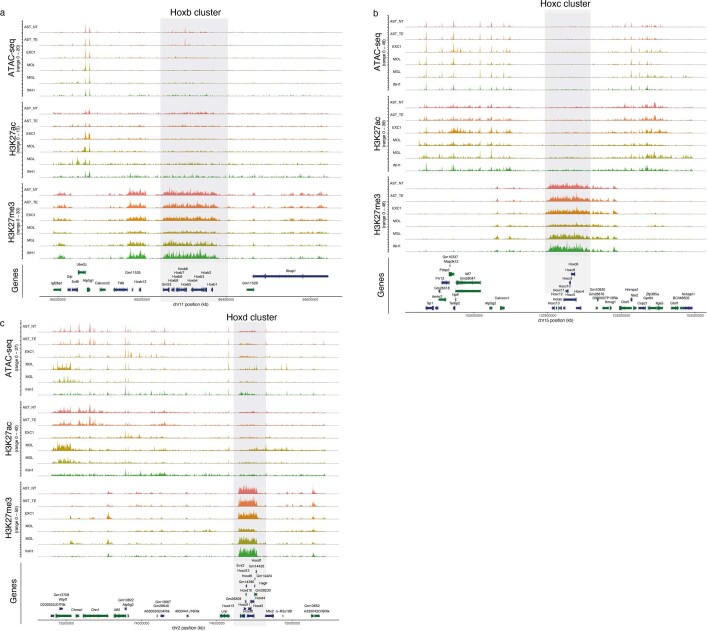
Genome browser tracks at Hox regions. **a**. Genome browser tracks for all modalities profiled by multimodal nano-CT (ATAC, H3K27ac, H3K27me3) around the **a**. HoxB, **b**. HoxC and **c**. HoxD loci.

To validate the specificity of individual deconvoluted modalities, we investigated chromatin states at the *Hox* genes clusters, expressed in the caudal but not rostral central nervous system, and found strong enrichment of H3K27me3, but not ATAC and H3K27ac, at *HoxA* locus (Fig. 4a), underscoring the specificity of individual modalities at the cluster level. Similar enrichment of H3K27me3 was observed in other Hox loci including *HoxB*, *HoxC*, and *HoxD* (Extended Data Fig. 5a–c). Then we performed principal component analysis (PCA) of ATAC-seq, H3K27ac, and H3K27me3 pseudo-bulk tracks for each individual cluster identified by scCUT&Tag^15^ (single modality) and nano-CT (multimodal). We selected the 50 most significant marker regions (peaks) from all clusters and across all modalities, merged any overlapping regions and used them as features for PCA. The PCA showed that cell populations identified through the individual modalities co-clustered together regardless of the method used for obtaining the data (Fig. 4b). We then selected a set of highly specific peaks for both H3K27ac and H3K27me3 on the basis of our previous scCUT&Tag data in astrocytes^15^ and generated a metagene plot of H3K27ac/H3K27me3 signal obtained by multimodal nano-CT. We observed high enrichment of the respective modifications only in the respective set of marker peaks (Fig. 4c). We also plotted correlation matrix of H3K27me3/H3K27ac signal in all peak regions identified in nano-CT and observed strong correlation of the respective H3K27ac and H3K27me3 tracks, and no correlation in H3K27ac–H3K27me3 combinations (Fig. 4d). Despite the high enrichment of the respective H3K27ac and H3K27me3 modalities, a small subset of peak regions showed signal of both H3K27ac and H3K27me3 specifically in nano-CT dataset (Fig. 4c). To further quantify this overlap, we intersected peaks called from H3K27ac and H3K27me3 in the astrocytes telencephalon cluster and found that 1,093 peaks were overlapping, representing 4.4% of H3K27ac peaks and 11.5% of H3K27me3 peaks (Fig. 4e).

**Extended Data Fig. 6:**
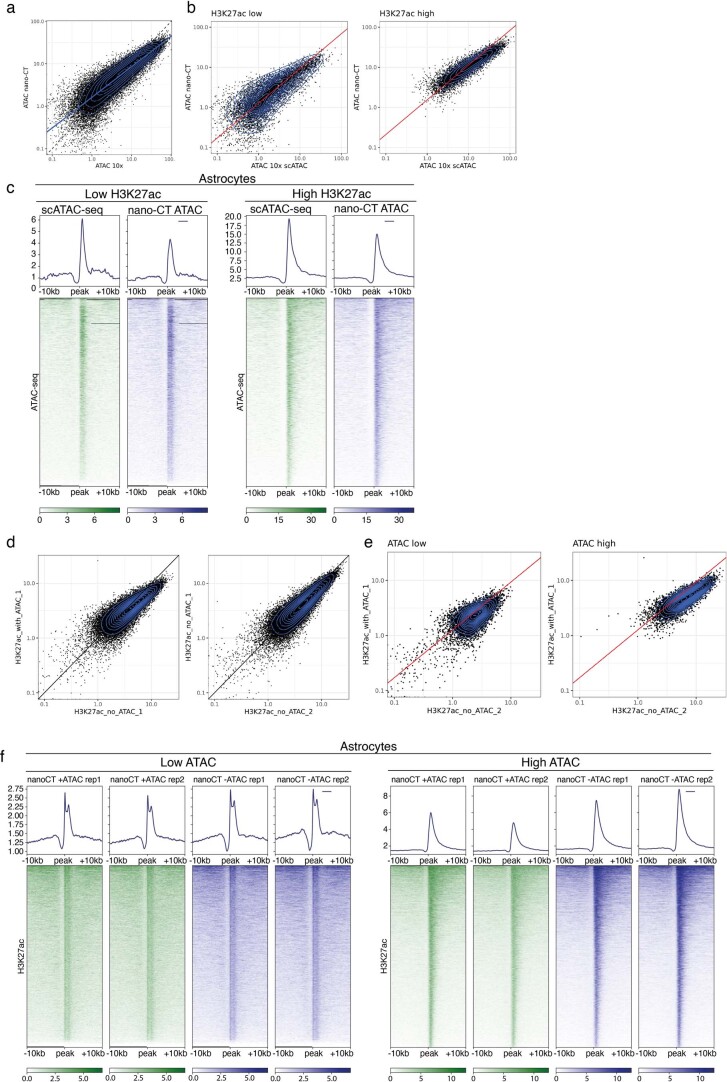
QC of cross-talk between the profiled modalities. **a**. Scatter plot showing the signal of scATAC-seq (single modality profiled. from 10x Genomics) and multimodal ATAC-seq performed with nano-CT in cluster astrocytes and in astrocyte-specific peaks. **b**. The same scatter plot as in **a**.,but stratified by H3K27ac signal. H3K27ac low represents peaks with the 20% lowest quantile of H3K27ac signal and H3K27ac high represents peaks with 20% highest quantile of H3K27ac signal. **c**. Metagene heatmaps showing the genomic distribution of the ATAC-seq signal in the same regions as in **b**. **d**. Scatter plot showing the cluster astrocytes H3K27ac signal profiled either together with H3K27me3 (without_ATAC) or together with ATAC-seq and H3K27me3 (with ATAC) within the astrocyte-specific peaks. **e**. The scatter plot of H3K27ac astrocytic signal within peak regions stratified by ATAC-seq signal. ATAC low represents peaks with the 20% lowest quantile of ATAC-seq signal and ATAC high represents peaks with the 20% highest quantile of ATAC-seq signal. **f**. Metagene heatmaps showing the genomic distribution of H3K27ac signal in the same regions as in **e**.

We also investigated how the overlapping modalities such as ATAC-seq and H3K27ac would interfere with each other. ATAC-seq tagmentation is performed as the first and optional step in the nano-CT protocol (Fig. 1c) and therefore we hypothesized that ATAC-seq signal should not be affected by other histone modalities. Indeed, the open chromatin signal obtained in nano-CT correlated well with the scATAC-seq profiled as single modality on the chromium platform (10x Genomics datasets) regardless of the levels of H3K27ac signal within the same region in astrocytes (Extended Data Fig. 6a–c). On the other hand, H3K27ac signal was slightly affected by the overlapping ATAC-seq signal specifically in regions with high levels of open chromatin and hence high ATAC-seq signal (Extended Data Fig. 6d–f). In summary, single-cell nano-CT can be used to simultaneously obtain robust and specific multimodal epigenetic profiles of several histone modifications and open chromatin from single cells.

### Integrative multimodal analysis of the epigenomic states

**Extended Data Fig. 7:**
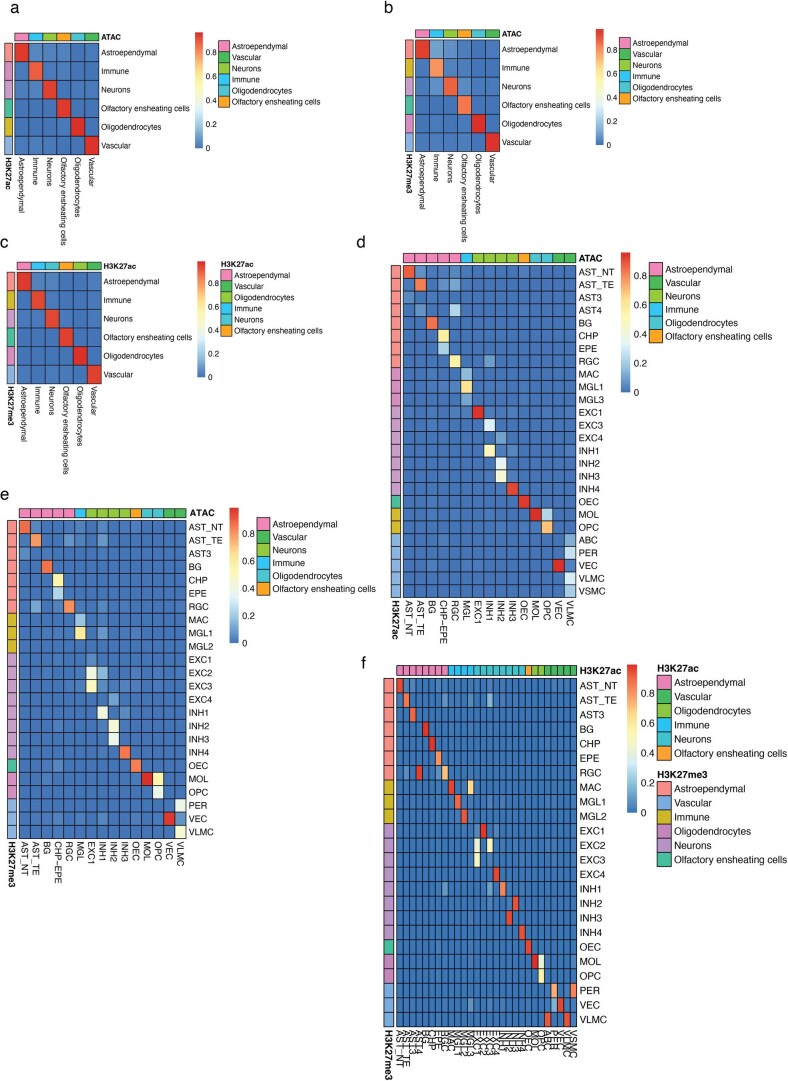
QC of cell identities across clusters in individual modalities. Confusion matrix of broad cell identities between **a**. ATAC and H3K27ac **b**. ATAC and H3K27me3 and **c**. H3K27ac and H3K27me3. Confusion matrices for fine cluster identities across the different modalities for **d**. ATAC and H3K27ac **e**.ATAC and H3K27me3 and **f**. H3K27ac and H3K27me3.

The variability in the number of identified clusters suggests that some modalities might be more informative towards cell identity, given the similar number of fragments per cell (Fig. 2b). To investigate whether the cell identities assigned using different modalities concord, we generated a confusion matrix for all combinations of modalities (Extended Data Fig. 7). The major cell type identities were fully recapitulated across all three modalities (Extended Data Fig. 7a–c), whereas there were specific clusters identified only in subset individual modalities (Extended Data Fig. 7d–f). For example, pericytes and vascular smooth muscle cells can be deconvoluted from the H3K27ac modality, but not from the H3K27me3 or ATAC-seq modality (Extended Data Fig. 7d–f).

**Fig. 5:**
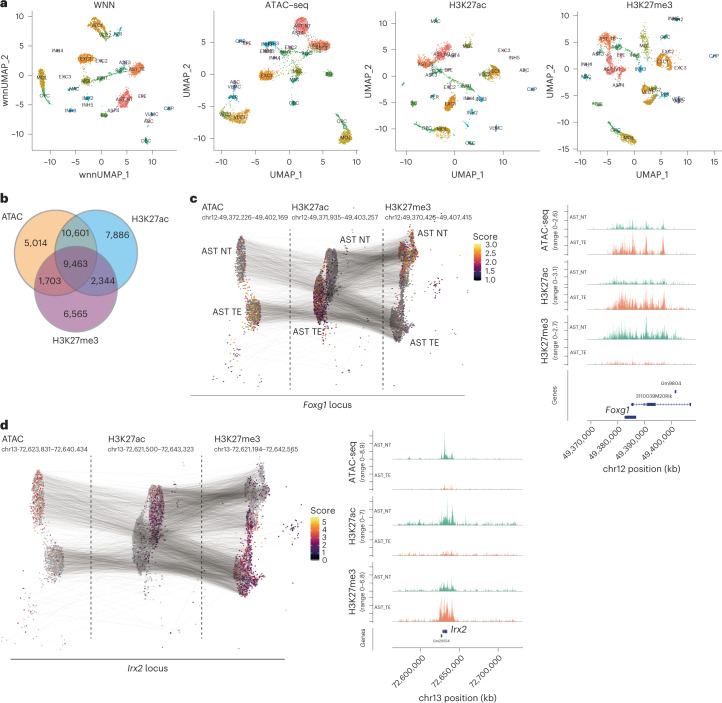
Multimodal analysis and visualization of the nano-CT data. **a**, UMAP embedding of the individual modalities with cluster labels identified through WNN analysis. Embedding is based on individual modalities, whereas cluster identities are assigned from WNN dimensionality reduction. **b**, Venn diagram showing the overlap of peaks identified from the individual modalities. **c**,**d**, UMAP projection and visualization of ATAC, H3K27ac and H3K27me3 signal intensity in single cells at the *Foxg1* (**c**) and *Irx2* loci (**d**). Gray lines connect the cells with same the single-cell barcodes across the different modalities. Clusters for telencephalon astrocytes (AST_TE) and non-telencephalon astrocytes (AST_NT) were selected for the visualization. Aggregated pseudo-bulk tracks for all modalities together with genomic annotations are shown to the right.

**Extended Data Fig. 8:**
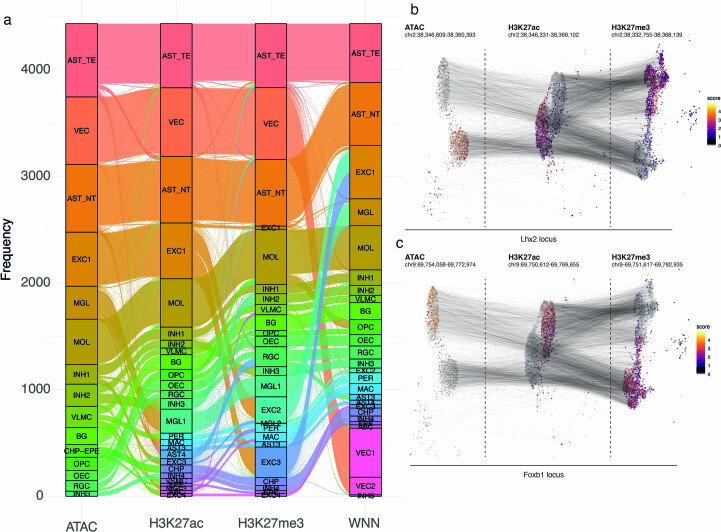
QC of cell identities and markers across clusters in individual modalities. **a**. Alluvial diagram of corresponding cell identities between the ATAC, H3K27ac and H3K27me3 modalities. **b,c**. UMAP projection and visualization of ATAC, H3K27ac and H3K27me3 signal intensity in single cells at the *Lhx2* **(b)** and Foxb1 **(c)** locus. Gray lines connect the cells with same single-cell barcodes across the different modalities. Clusters telencephalon astrocytes (AST_TE) and non-telencephalon astrocytes (AST_NT) were selected for the visualization.

To further improve the clustering, we used all of the multimodal matrices to perform weighted nearest neighbors (WNN) analysis^35^. The WNN largely recapitulated the clusters identified by each individual modality (Fig. 5a and Extended Data Fig. 8a). We then investigated whether features that explain most of the variability in single cells (LSI component loadings) overlapped among the different modalities. We found that H3K27ac and the ATAC-seq features overlapped the most (10,601 overlapping regions), but also a large fraction of genomic regions showed variability in all three modalities simultaneously (9,463) (Fig. 5b). For example, the locus surrounding gene coding for transcription factor *Foxg1* was heavily regulated in two major classes of astrocytes. Whereas the chromatin surrounding *Foxg1* was primarily open and K27 acetylated in telencephalon astrocytes, it was strongly K27 trimethylated in non-telencephalon astrocytes (Fig. 5c). Given that Foxg1 has previously been linked with early neurogenesis in the forebrain^36^ and with inhibition of gliogenesis^37,38^ the epigenomic state of astrocytes likely reflects the developmental origin of the astrocyte populations. Other developmental and patterning transcription factors such as *Lhx2*, *Foxb1* or *Irx2* were also found to be differentially enriched at an epigenomic level in astrocyte populations (Fig. 5d and Extended Data Fig. 8b,c).

### Sequential waves of H3K27me3 in the oligodendrocyte lineage

**Fig. 6:**
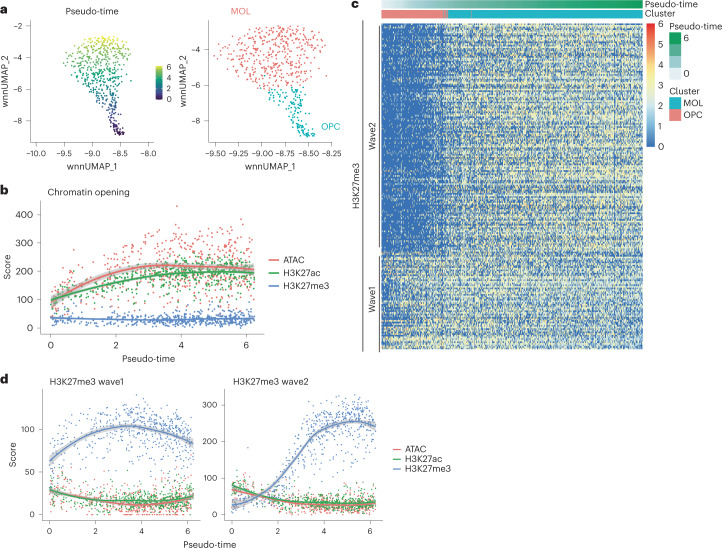
nano-CT reveals sequential H3K27me3 waves during oligodendrocyte differentiation. **a**, UMAP embedding showing pseudo-time calculated by slingshot on the basis of WNN dimensionality reduction and cluster identities. **b**, Scatter plot depicting meta-region score for all modalities (*y*-axis) and pseudo-time (*x*-axis). The score was calculated as a sum of normalized score across all regions. The regions were selected on the basis of *P* value (*P* < 0.05, Wilcoxon test) and log fold change > 0 at the marker regions of the ATAC modality, and top 200 regions were used. The line depicts local polynomial regression fit (loess) of the data and shaded regions depict 95% confidence intervals. **c**, Heat map representation of the H3K27me3 signal intensity at the regions the marker regions that are gaining H3K27me3 during oligodendrocytes differentiation (*P* < 0.05, Wilcoxon test, log fold change > 0, top 200 regions). Each column depicts one single cell and row single genomic region (peak). Cells are ordered by pseudo-time calculated as shown in **a**. The order of the regions is based on *k*-means clustering of the matrix with *k* = 2. **d**, Scatter plots depicting meta-region score for all modalities (*y*-axis) and pseudo-time (*x*-axis). The score was calculated as a sum of normalized score across all regions. The regions were selected on the basis of *P* value (*P* < 0.05, Wilcoxon test) and log fold change > 0 at the marker regions of the H3K27ac modality, and top 200 regions were used. The regions were further stratified to wave 1 and wave 2 regions on the basis of *k*-means clustering as shown in **c**. The line depicts local polynomial regression fit (loess) of the data and shaded regions depict 95% confidence intervals.

**Extended Data Fig. 9:**
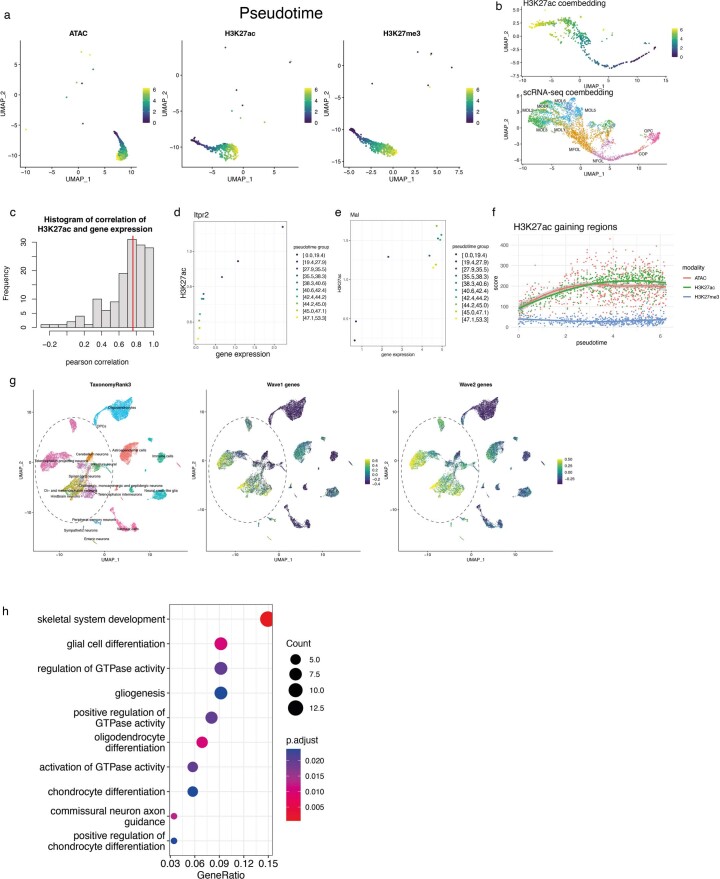
Multimodal pseudotime and chromatin velocity. **a**. UMAP embedding and visualization of pseudotime determined based on the WNN dimensionality reduction. Pseudotime is projected on UMAPs generated by dimensionality reduction of the individual modalities. **b**. UMAP representation of co-embedded H3K27ac nano-CT dataset and scRNA-seq dataset^41^. Diffusion pseudotime calculated by scvelo is projected onto the H3K27ac UMAP. **c**. Histogram of correlation of gene expression and H3K27ac across binned, integrated pseudotime categories. **d, e** Phase plot of H3K27ac and gene expression across binned, integrated pseudotime for **d**. *Itpr2* and **e**. *Mal*. **f**. Scatter plot depicting meta-region score for all modalities (y-axis) and pseudotime (x-axis). The score was calculated as a sum of normalized score across all regions. The regions were selected based on p-value (p < 0.05, Wilcoxon test) and log fold change > 0 at the marker regions of the H3K27ac modality, and top 200 regions were used. The line depicts local polynomial regression fit (loess) of the data and shaded regions depict 95% confidence intervals **g**. UMAP embedding of the scRNA-seq data from the mouse brain atlas^34^. 20,000 single cells were sampled randomly from the dataset. Wave 1 and wave2 scores were calculated as a mean of scaled gene expression data for genes identified in Fig. 6c. **h**. Gene ontology enrichment analysis of genes, which are repressed in the wave 2 of H3K27me3-mediated repression. P value was calculated using R package enrichGO, using one sided Fischer’s test with Benjamini-Hochberg correction for multiple hypothesis testing.

One of the strengths of multimodal nano-CT is that it allows for direct and simultaneous analysis of the dynamics of multiple histone marks and chromatin accessibility in the same cells. Our post-natal day P19 brain dataset covers the progression of the whole oligodendrocyte lineage from oligodendrocyte progenitor cells towards mature oligodendrocytes. Therefore, we focused on the oligodendrocyte lineage, and we used the combined WNN embedding to generate pseudo-time of oligodendrocyte differentiation (Fig. 6a). We then projected the pseudo-time identified from WNN onto UMAPs for the individual modalities, which recapitulated the gradient found in WNN embedding (Extended Data Fig. 9a). Moreover, the predicted trajectory was consistent with the published trajectory of oligodendrocytes differentiation identified by scRNA-seq (Extended Data Fig. 9b) and gene expression and H3K27ac was correlated for lineage marker genes (Extended Data Fig. 9c–e).

Then, we investigated the magnitude of changes in all measured chromatin modalities at sites that are the most dynamically opening or acquiring histone modifications. We observed that loci that are opening the chromatin (ATAC-seq) acquire H3K27ac with a slight pseudo-time delay, whereas there is overall little change in the overall H3K27me3 state of these sites (Fig. 6b). The ATAC signal reaches its plateau in an earlier stage in the pseudo-time than H3K27ac for the top open chromatin regions. A similar, although less prominent effect is observed, when looking specifically at loci that acquire H3K27ac during oligodendrocytes differentiation (Extended Data Fig. 9f). Thus, our analysis indicates that the chromatin opening precedes deposition of H3K27ac at loci that are marked for gene expression.

Strikingly, we found that H3K27me3 deposition occurs in two distinct waves during oligodendrocytes differentiation (Fig. 6c,d). The first wave of H3K27me3 occurred early on in the differentiation process and was associated with repression of genes expressed predominantly in neurons, whereas the second wave repressed both neuronal genes (Extended Data Fig. 9g) and genes expressed in oligodendrocyte progenitor cells (for example *Sox5*, *Sox6*, and *Ptprz1*), which are associated with Gene Ontology (GO) terms gliogenesis, glial cell differentiation, and oligodendrocyte differentiation (Extended Data Fig. 9h). Thus, oligodendrocyte lineage progression encompasses two sequential H3K27me3 repressive states, which would not be possible to discriminate using transcriptomic data. In summary, multimodal nano-CT analysis allows unique insights into the epigenomic processes driving biological processes such as oligodendrocyte differentiation.

### Chromatin velocity of oligodendrocyte lineage

**Fig. 7:**
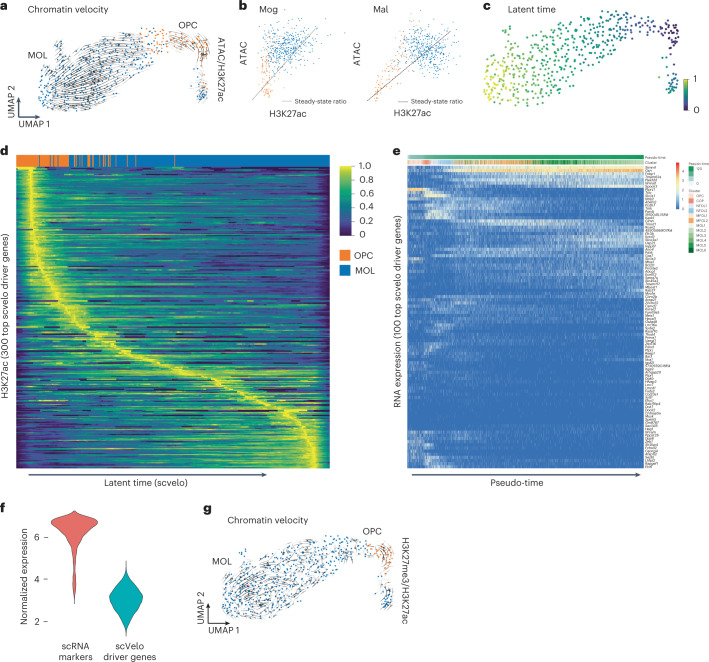
nano-CT-based chromatin velocity analysis. **a**, UMAP projection and chromatin velocity visualization. The chromatin velocity was calculated by using ATAC-seq gene-by-cell matrix as input into the unspliced layer and H3K27ac gene-by-cell matrix into the spliced layer and then running scvelo algorithm using default parameters. **b**, Phase plots of ATAC-seq and H3K27ac signal for key genes associated with oligodendrocyte differentiation (*Mal*, *Mag*). **c**, UMAP projection of the latent time calculated by the scvelo algorithm. **d**, Heat map showing H3K27ac signal normalized with sctransform^42^. Rows depict individual top velocity driver genes, sorted by time of value with maximum intensity and columns depict individual cells sorted by latent time. **e**, Heat map representing gene expression profiles measured by scRNA-seq^41^ in the oligodendrocyte lineage. Rows depicts individual genes, clustered by similarity and columns depict single cells ordered in pseudo-time. **f**, Violin plot showing normalized expression of set of marker genes identified in scRNA-seq dataset, and normalized expression of a set of genes identified as the key driver genes by scvelo. **g**, UMAP projection and velocity vectors projection of chromatin velocity calculated using H3K27ac gene-by-cell matrix used as input into unspliced layer and H3K27me3 gene-by-cell matrix used as input into the spliced layer and then running the scvelo algorithm using default parameters.

It has been shown that differentiation kinetics can be predicted from scRNA-seq data using RNA velocity modeling on the basis of the ratio of spliced/unspliced mRNA^39^. Recently, a similar concept has been proposed for chromatin velocity, using information from two anti-correlated chromatin modalities—heterochromatin and open chromatin^29^. Our multimodal chromatin profiling allows us to define several chromatin velocities: ATAC/H3K27ac; ATAC/H3K27me3; and H3K27ac/H3K27me3. Our analysis (Fig. 6b) suggests that chromatin accessibility precedes H3K27ac, which is analogous to the spliced/unspliced mRNA relationship. Although the difference between ATAC-seq and H3K27ac is subtle, it led us to hypothesize that we can directly leverage the velocity framework to predict the directionality of a differentiation pathway. We tested this idea by generating a gene-by-cell matrix for ATAC and H3K27ac and using these as an input into the scvelo algorithm^40^. Indeed, ATAC/H3K27ac velocity accurately predicted the differentiation trajectory of the oligodendrocyte lineage from oligodendrocyte progenitor cells towards mature oligodendrocytes (Fig. 7a). Phase plots of ATAC-seq versus H3K27ac of several genes associated with oligodendrocyte differentiation such as *Mal* or *Mog* further supported this directionality (Fig. 7b).

**Extended Data Fig. 10:**
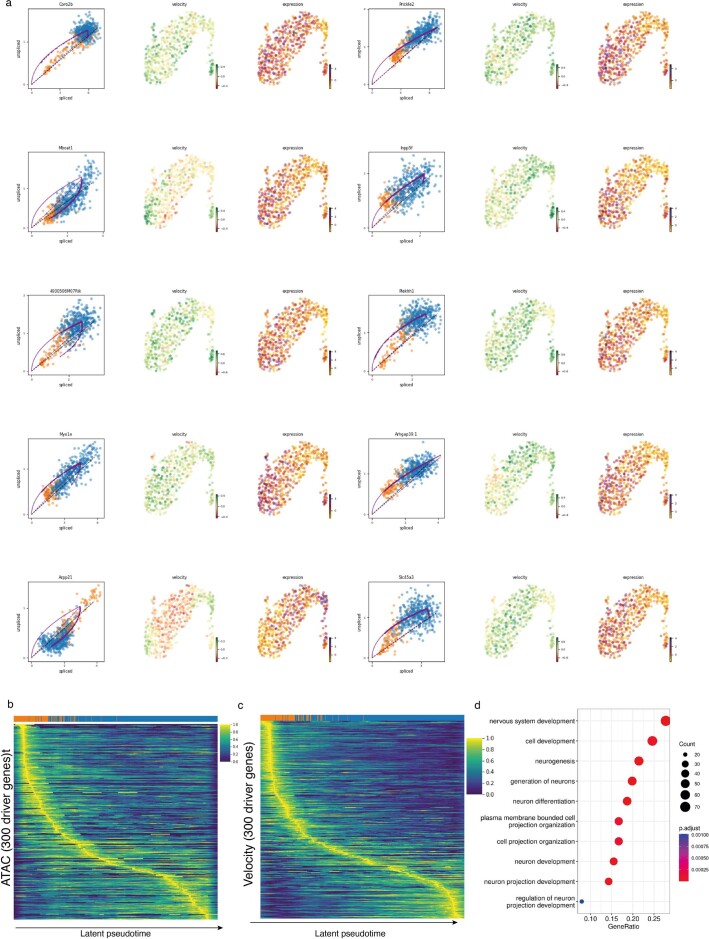
Chromatin velocity inference from nano-CUT&Tag data. **a**. Phase plots of H3K27ac and ATAC, velocity and expression signal for top 10 most likely velocity driver genes identified by scvelo. **b**. Heatmap showing ATAC-seq signal normalized with sctransform^42^. Rows depict individual top velocity driver genes, sorted by time of value with maximum intensity and columns depict individual cells sorted by latent time. **c**. Heatmap showing the chromatin velocity. Rows depict individual top velocity driver genes, sorted by time of value with maximum intensity and columns depict individual cells sorted by latent time. **d**. Gene ontology enrichment analysis of the most important velocity driver genes. P value was calculated using R package enrichGO, using one sided Fischer’s test with Benjamini-Hochberg correction for multiple hypothesis testing.

We then identified the key velocity driver genes (Extended Data Fig. 10a and Supplementary Table 2) and plotted normalized H3K27ac, ATAC signal, and velocity along inferred latent time (Fig. 7c) highlighting the dynamic changes in chromatin during oligodendrocyte differentiation (Fig. 7d and Extended Data Fig. 10b,c). We validated that the majority of the driver genes also show variable gene expression (measured by scRNA-seq) in the oligodendrocyte lineage^41^ (Fig. 7e). Interestingly, these driver genes are relatively lowly expressed, compared to the typical marker genes identified by scRNA-seq (Fig. 7f). The GO terms associated with these genes were nervous system development, cell development, and neurogenesis or generation of neurons (Extended Data Fig. 10d), suggesting that the set of genes identified through chromatin velocity comprised non-canonical oligodendrocyte differentiation genes. This indicates the potential of multimodal chromatin profiling and chromatin velocity modeling in identification of genes, which might be dynamically regulated through changes in the chromatin landscape, but difficult to pick up through gene expression profiling.

Finally, we attempted to model RNA velocity using other combinations of chromatin modalities that do not follow the same causal relationship as ATAC/H3K27ac as input into the velocity analysis. H3K27ac/H3K27me3 are anti-correlated and mutually exclusive histone marks and this combination of modalities did not correctly predict the oligodendrocyte differentiation trajectory (Fig. 7g). Thus, anti-correlated active and repressive chromatin marks might require other modeling or data pre-processing strategies to correctly predict chromatin velocity.

## Discussion

While most of the current epigenomic single-cell technologies are unimodal (Supplementary Table 3), several studies have attempted to profile two epigenetic modalities at the same time through direct measurement^28,29^ or imputation from other co-profiled modality^20,23^. Profiling of three epigenetic modalities simultaneously has been previously achieved at the bulk level with Multi-CUT&Tag, suggesting that this technology might also allow this trimodal profiling at single-cell level. Here we describe nano-CT, a new multimodal scCUT&Tag protocol, with similarities to NTT-Seq^32^ which allows profiling thousands of single cells while (1) reducing the requirements for input material by around 5-fold; (2) increasing the number of fragments detected per cell by 16-fold compared to scCUT&Tag; and (3) allowing the simultaneous probing in each single cell of 3 epigenomic modalities—chromatin accessibility (ATAC-seq), two histone marks (H2K27ac and H3K27me3).

We applied nano-CT to the mouse juvenile brain demonstrating that the simultaneous detection of ATAC, repressive and active histone marks can provide further deconvolution of cell heterogeneity. Moreover, we found that individual modalities are not equally effective in the identification of cell identity and that histone marks can be more informative than, for example, open chromatin regions, given similar data quality. Finally, we show that nano-CT could provide direct measurement of time-resolved dynamics of chromatin enhancer activation, resolve distinct waves of repressive H3K27me3 and define chromatin velocity in single cells.

The new antibody and nano-Tn5 incubation strategy reduces the number of steps in CUT&Tag protocol compared to previous generation scCUT&Tag retaining more nuclei for analysis. One 10x chromium chip can be loaded with as little as 25,000 input nuclei, when profiling two histone modifications (nano-CT without ATAC), although 50,000 input nuclei would provide a more complex dataset (Fig. 1b). When profiling open chromatin together with histone post-translational modifications, open chromatin tagmentation is performed before nano-CT antibody incubations and thus an increased number of centrifugation and washing steps pushes the input requirements to roughly 200,000 nuclei. Nevertheless, nano-CT can with similar input requirements to scCUT&Tag profile up to three chromatin modalities at the same time.

Previous scCUT&Tag library construction protocols rely on two tagmentation events in close proximity and correct orientation for amplification of single fragment. This results in very high specificity of scCUT&Tag but also restricts the number of fragments per cell. By contrast, the nano-CT tagmentation strategy uses MeA-only-loaded Tn5 to deterministically tagment the chromatin. Linear amplification of the tagmented chromatin ensures that also events with single integration (orphan tagmentation) are pre-amplified and used for library construction. This increases the number of fragments per cell by more than tenfold, but also reduces slightly the specificity of the assay resulting in a lower fraction of reads in peak regions. Despite higher levels of background tagmentation, overall, nano-CT provides superior single-cell data quality for one-modality profiling compared to scCUT&Tag and enables multimodal chromatin profiling.

Integration of scCUT&Tag-Pro datasets has previously suggested that H3K27me3 repressive states might be more heterogeneous than what gene expression allows us to infer^31^. Here we show that multimodal nano-CT permits deconvolution of H3K27me3 states in a continuous and dynamic biological process such as oligodendrocyte differentiation. Sequential waves of H3K27me3 occur in pseudo-time, at distinct loci regulating distinct modules necessary for efficient oligodendrocyte lineage progression and differentiation.

Furthermore, the multimodal measurements of ATAC/H3K27ac/H3K27me3 led us to define chromatin velocity and predict dynamics of cell differentiation from a snapshot dataset. The ATAC/H3K27ac chromatin velocity builds upon the previously proposed concepts of RNA velocity^39,40^, chromatin potential of ATAC/RNA-seq^8^ and chromatin velocity of eu-/heterochromatin^29^. Nonetheless, the functional relationships between chromatin modalities differ from unspliced/spliced RNA and therefore other modeling approaches with different assumptions should be specifically tailored to chromatin velocities defined by their specific modalities. Development of novel computational methods that can tackle these relationships will undoubtedly be necessary to capture the full predictive potential of multimodal datasets. Moreover, measurements of more than three chromatin modalities or chromatin and gene expression from single cells, by expanding our fusion nanobody approach to other species or using primary nanobodies, will provide data that can drive more accurate predictive algorithms and models to more reliably assign functional genomic elements to their target genes.

nano-CT and NTT-Seq are the latest of a new series of multimodal single-cell epigenomic technologies that also includes Multi-CUT&Tag and scGET-seq. These technologies have specific advantages and disadvantages which include fragments per cell, input material, and throughput, among other parameters (Supplementary Table 3), which might make them more amenable for application in specific biological contexts then others. Looking forward, the use of fusion proteins of Tn5 with single-chain nanobodies recognizing mouse and rabbit antibodies confers a unique versatility to nano-CT. The modular design of the presented nano-Tn5 fusions permit adopting this strategy to virtually any combination of histone marks or chromatin-binding proteins. Tn5 fusion with other single-chain nanobodies might allow analyzing more than three epigenomic modalities in the same cell, at the same time, which is bound to provide further insights into chromatin dynamics. We anticipate that measurement of levels of other histone marks, or chromatin-binding factors (transcription factors, chromatin remodelers, RNA polymerase etc.) with nano-CT can provide mechanistic insights into multitude of processes such as activation of enhancers, promoters, initiation of transcription, or bivalency.

## Methods

A detailed step by step protocol for nano-CT is available on protocols.io (https://www.protocols.io/view/nano-cut-amp-tag-for-multimodal-profiling-of-the-c-8epv59o8dg1b/v2). A detailed list of required reagents is in Supplementary table 4.

### Animals

The mouse line used in this study was generated by crossing Sox10:Cre animals^7^ (The Jackson Laboratory mouse strain 025807) on a C57BL/6j genetic background with RCE:loxP (EGFP) animals (The Jackson Laboratory mouse strain 32037-JAX) on a C57BL/6xCD1 mixed genetic background. Females with a hemizygous Cre allele were mated with males lacking the Cre allele, while the reporter allele was kept in hemizygosity or homozygosity in both females and males. In the resulting Sox10:Cre-RCE:LoxP (EGFP) animals the entire oligodendrocyte lineage was labeled with EGFP. Breeding with males containing a hemizygous Cre allele in combination with the reporter allele to non-Cre carrier females resulted in offspring where all cells were labeled with EGFP and was therefore avoided. All animals were free from mouse viral pathogens, ectoparasites and endoparasites, and mouse bacteria pathogens. Mice were kept with the following light/dark cycle: dawn 6:00–7:00, daylight 7:00–18:00, dusk 18:00–19:00, night 19:00–6:00 and housed to a maximum number of five per cage in individually ventilated cages (IVC sealsafe GM500, tecniplast). General housing parameters such as relative humidity, temperature, and ventilation follow the European convention for the protection of vertebrate animals used for experimental and other scientific purposes treaty ETS 123, Strasbourg 18.03.1996/01.01.1991. In brief, consistent relative air humidity of 55 ± 10%, 22 °C and the air quality is controlled with the use of standalone air-handling units supplemented with HEPA-filtrated air. Monitoring of husbandry parameters is done using ScanClime (Scanbur) units. Cages contained hardwood bedding (TAPVEI, Estonia), nesting material, shredded paper, gnawing sticks, and card box shelter (Scanbur). The mice received regular chow diet (either R70 diet or R34, Lantmännen Lantbruk). Water was provided by using a water bottle, which was changed weekly. Cages were changed every other week. All cage changes were done in a laminar air-flow cabinet. Facility personnel wore dedicated scrubs, socks, and shoes. Respiratory masks were used when working outside of the laminar air-flow cabinet. Animals were sacrificed at juvenile stages (P19) and both sexes were included in the study. All experimental procedures on animals were performed following the European directive 2010/63/EU, local Swedish directive L150/SJVFS/2019:9, Saknr L150 and Karolinska Institutet complementary guidelines for procurement and use of laboratory animals, Dnr. 1937/03–640. The procedures described were approved by the local committee for ethical experiments on laboratory animals in Sweden (Stockholms Norra Djurförsöksetiska nämnd), license number 144/16, 1995_2019 and 7029/2020.

### Antibodies

The following antibodies were used in the multimodal nano-CT experiments: mouse anti-H3K27me3 (Abcam, Ab6002), rabbit anti-H3K27ac (Abcam, Ab177178). Anti-H3K27me3 (Cell Signaling, 9733T) was used in the single-modality nano-CT experiment.

### Nanobody-Tn5 fusion design and production

The sequence of secondary nanobodies was taken from Pleiner et al.^30^ and fused to the sequence of hyperactive Tn5 transposase. For anti-rabbit-Tn5 fusion with nanobody TP897 and for anti-mouse-Tn5 fusion with nanobody TP1170 were designed. The full sequence of the fusion proteins can be found in Supplementary Sequences 1 and 2. The full-length fusion protein sequences were ordered from Twist Bioscience cloned into twist expression vector. The constructs were transformed into BL21 (DE3) Star *Escherichia coli*, inoculated into an overnight culture and grown in TB medium supplemented with 8 g l^−1^ glycerol at 30 °C with 175 r.p.m. shaking in the presence of 0.4% glucose. The next day the cultures were grown in the LEX system starting in the morning with cultivation at 37 °C. The optical density was measured at different times and the temperature was switched to 18 °C, when the culture reached an optical density of 2. The protein expression was induced at an approximate optical density of 3 (isopropyl β-d-1-thiogalactopyranoside, final concentration 0.5 mM). Protein expression continued overnight before the cells were collected by centrifugation (10 min at 4,500*g*). IMAC lysis buffer (1.5 ml buffer per gram cell pellet) and the complete stock solution (1 tablet Complete EDTA-free (protease inhibitor cocktail, Roche) and 50 μl benzonase nuclease cell resuspension (PSF) per 1 ml; 1 ml per 1.5 l culture) were added and the cell pellets were resuspended on a shaker table (cold room). The resuspended cell pellets were stored at −80 °C.

The frozen cell pellets were thawed, extra benzonase was added and the cells were disrupted by pulsed sonication (4 s/4 s 4 min, 80% amplitude). The sonicated lysates were centrifuged (20 min at 49,000*g*) and the soluble fractions were decanted and filtered through 0.45-μm filters. The samples were loaded onto the ÄKTA Xpress and purified overnight.

The following buffers were used: lysis buffer (100 mM HEPES, 500 mM NaCl, 10% glycerol, 10 mM imidazole, 0.1 mM EDTA, 0.1% Triton X-100, 0.5 mM TCEP, pH 8.0); wash 1 buffer (20 mM HEPES, 500 mM NaCl, 10% glycerol, 10 mM imidazole, 0.1 mM EDTA, 0.1% Triton X-100, 0.5 mM TCEP, pH 7.2); wash 2 buffer (20 mM HEPES, 1000 mM NaCl, 10% glycerol, 50 mM imidazole, 0.1 mM EDTA, 0.1% Triton X-100, 0.5 mM TCEP, pH 7.2); elution buffer (20 mM HEPES, 500 mM NaCl, 10% glycerol, 500 mM imidazole, 0.1 mM EDTA, 0.1% Triton X-100, 0.5 mM TCEP, pH 7.2); gel filtration buffer (50 mM HEPES, 500 mM NaCl, 10% glycerol, 0.1 mM EDTA, 0.1% Triton X-100, 1 mM dithiothreitol (DTT), pH 7.2); final batch buffer (50 mM HEPES, 500 mM NaCl, 50% glycerol, 0.1 mM EDTA, 0.1% Triton X-100, 1 mM DTT, pH 7.2); IMAC column (5 ml HisTrap HP (GE Healthcare)); gel filtration column (HiLoad 16/60 Superdex 200 (GE Healthcare)). Selected fractions were examined on SDS-PAGE gels before pooling. Fractions containing the target proteins were pooled and concentrated with Vivaspin concentration filters (Vivascience, 50 kDa cut off) at 4,000*g*. The samples were then diluted with buffer containing 60% glycerol to get 50% in the final buffer. The final concentration was measured by absorbance at 280 nm (Nanodrop), the protein was flash frozen in aliquots of 200 μl in liquid nitrogen and stored at −80 °C.

### Tn5 loading

Nanobody-Tn5 fusion protein was loaded with barcoded oligonucleotides. First, an equimolar mixture of 100 μM forward oligonucleotides (Tn5_P5_MeA_BcdX_0N, Tn5_P5_MeA_BcdX_1N, Tn5_P5_MeA_BcdX_2N, Tn5_P5_MeA_BcdX_3N; Supplementary Table 1) was mixed (5 μl each) with an equimolar amount of 100 μM of Tn5_Rev oligonucleotide (20 μl). The oligonucleotide mixture was denatured for 5 min at 95 °C and annealed by slowly ramping down the temperature at 0.1 °C s^−1^ in a thermocycler. The nano-Tn5 was loaded by mixing the following: 8 μl annealed (P5) oligonucleotides, 42 μl glycerol, 44.1 μl 2× dialysis buffer (100 mM HEPES-KOH pH 7.2, 200 mM NaCl, 0.2 mM EDTA, 2 mM DTT (add fresh), 0.2% Triton X, 20% glycerol) 5.9 μl anti-mouse-nano-Tn5 (5 mg ml^−1^, 67.6 μM) or 8 μl annealed oligonucleotides, 42 μl glycerol, 45.7 μl 2× dialysis buffer (100 mM HEPES-KOH pH 7.2, 200 mM NaCl, 0.2 mM EDTA, 2 mM DTT, 0.2% Triton X, 20% glycerol) 4.3 μl anti-rabbit-nano-Tn5 (6.8 mg ml^−1^, 93 μM) to get final 2 μM loaded nano-Tn5 dimer.

Non-fused Tn5 (as in ATAC-seq) was loaded using Tn5_P7_MeB standard oligonucleotides (referred to as P7 Tn5). First, the 100 μM Tn5_P7_MeB oligonucleotides was mixed with an equimolar amount of 100 μM Tn5_Rev. The oligonucleotide mixture was denatured for 5 min at 95 °C and annealed by slowly ramping down the temperature at 0.1 °C per second in a thermocycler. The Tn5 was loaded by mixing the following: 8 μl annealed oligonucleotides (P7); 43.12 μl glycerol; 42.6 μl 2× dialysis buffer (100 mM HEPES-KOH pH 7.2, 200 mM NaCl, 0.2 mM EDTA, 2 mM DTT (add fresh), 0.2% Triton X, 20% glycerol); and 6.28 μl Tn5 (3.5 mg ml^−1^, 63.64 μM).

### Tissue dissociation

Mice were sacrificed, perfused with aCSF buffer (87 mM NaCl, 2.5 mM KCl, 1.25 mM NaH_2_PO_4_, 26 mM NaHCO_3_, 75 mM sucrose, 20 mM glucose, 0.5 mM CaCl_2_, and 4 mM MgSO_4_) and brain was removed. The brain was dissociated into a single-cell suspension using the Neural Tissue Dissociation Kit P (Miltenyi Biotec, 130-092-628) according to the manufacturer’s protocol. For mice older than P7, myelin was removed using debris removal solution (Miltenyi Biotec, 130-109-398) according to the manufacturer’s instructions. The single-cell suspension was filtered through a 50-μm cell strainer.

### nano-CT

Two-hundred thousand cells (nano-CT without ATAC-seq) were centrifuged for 5 min at 500*g*, resuspended in 200 μl antibody buffer (20 mM HEPES pH 7.5, 150 mM NaCl, 2 mM EDTA, 0.5 mM spermidine, 0.05% digitonin, 0.01 % NP-40, 1× protease inhibitors, and 2% bovine serum albumin (BSA)) and incubated for 3 min on ice to extract nuclei. Nuclei were then centrifuged at 600*g* for 3 min and resuspended in 100 μl antibody buffer pre-mixed with 1:100 diluted primary mouse antibody, 1:100 diluted primary rabbit antibody, 1:100 diluted anti-rabbit-nano-Tn5 loaded with barcoded P5 oligonucleotide (2μM stock), and 1:100 diluted anti-mouse-nano-Tn5 (2μM stock) loaded with a different barcoded P5 oligonucleotide. The barcodes that were used were recorded. The sample was then incubated at 4 °C on a roller overnight.

After overnight incubation, the nuclei were centrifuged at 600*g* for 3 min and washed twice with Dig-300 buffer (20 mM HEPES pH 7.5, 300 mM NaCl, 0.5 mM spermidine, 0.05% digitonin, 0.01% NP-40, 2% BSA, and 1× protease inhibitors). After that, the nuclei were resuspended by pipetting in 200 μl tagmentation buffer (20 mM HEPES pH 7.5, 300 mM NaCl, 0.5 mM spermidine, 0.05% digitonin, 0.01% NP-40, 2% BSA, 1× protease inhibitors, and 10 mM MgCl_2_) and incubated for 1 h at 37 °C. The sample was mixed by pipetting after 30 min of the incubation to prevent sedimentation. The reaction was stopped by addition of 200 μl 1× diluted nuclei buffer (Chromium Next GEM Single Cell ATAC Library and Gel Bead Kit v1.1; 10x Genomics) supplemented with 2% BSA (1× DNB/BSA) and 12.5 mM EDTA. The nuclei were sedimented by centrifugation at 600*g* for 3 min and washed twice with 200 μl 1× DNB supplemented with 2% BSA. After the second wash and centrifugation, around 180 μl 1× DNB/BSA supernatant was removed from the sample. The nuclei were resuspended in the remaining 20 μl 1× DNB/BSA, 2 μl were mixed with 8 μl trypan blue and counted manually using a counting chamber. Sixteen thousand nuclei were used to load the 10x chromium chip. Nano-CT without ATAC was performed in two biological replicates and, in parallel, nano-CT with ATAC was performed on the same biological sample as a technical replicate.

### nano-CT with ATAC-seq

ATAC-seq was performed as described in ref. ^43^ (Omni-ATAC) with minor modifications. Tn5 was loaded with barcoded MeA/Me-Rev oligonucleotides omitting the MeB/MeR (see above and Supplementary Table 1). The number of cells used as input was 200,000. The cells were counted, centrifuged for 10 min at 300*g*, resuspended at 4 °C in 200 μl ATAC resuspension buffer (ARB, 10 mM Tris pH 7.5, 10 mM NaCl, 3 mM MgCl_2_ supplemented with 0.1% NP-40, 0.1% Tween-20 and 0.01% digitonin), pipetted up and down three times and incubated on ice for 3 min. Then 1 ml ATAC wash buffer (ARB, supplemented with 0.1 Tween-20) was added, and mixed by inverting the tube three times. Nuclei were pelleted by centrifugation at 500*g* for 10 min at 4 °C on swinging bucket rotor centrifuge with appropriate adapters for microtubes. Supernatant was aspirated and cell pellet as resuspended in 200 μl transposition mix (100 μl 1× TD buffer, 66 μl 1× PBS, 2 μl 10% Tween-20, 2 μl 1% digitonin, 10 μl of 2μM Tn5 transposase loaded with barcoded MeA/Rev (P5) oligonucleotides, 20 μl water), pipetted up and down five times gently to mix and incubated at 37 °C for 30 min with 1,000 r.p.m. mixing. After the tagmentation was finished, the nuclei were centrifuged at 500*g* for 10 min, supernatant was removed, and nuclei were washed with 200 μl CT antibody buffer (20 mM HEPES pH 7.5, 150 mM NaCl, 2 mM EDTA, 0.5 mM spermidine, 0.05% digitonin, 0.01 % NP-40, 1× protease inhibitors, and 2% BSA). The nuclei were then pelleted by centrifugation for 3 min at 600*g*, the washed again with 200 μl antibody buffer and centrifuged for 3 min at 600*g*. The pellet was then resuspended in 100 μl antibody buffer pre-mixed with 1:100 diluted primary mouse antibody, 1:100 diluted primary rabbit antibody, 1:100 diluted anti-rabbit-nano-Tn5 (2μM stock) loaded with barcoded P5 oligonucleotide and 1:100 diluted anti-mouse-nano-Tn5 (2μM stock) loaded with a different barcoded P5 oligonucleotide. The barcodes that were used were recorded. The sample was then incubated at 4 °C on a roller overnight.

After overnight incubation, the nuclei were centrifuged at 600*g* for 3 min and washed twice with Dig-300 buffer (20 mM HEPES pH 7.5, 300 mM NaCl, 0.5 mM spermidine, 0.05% digitonin, 0.01 % NP-40, 2% BSA, and 1× protease inhibitors). After that, the nuclei were resuspended by pipetting in 200 μl tagmentation buffer (20 mM HEPES pH 7.5, 300 mM NaCl, 0.5 mM spermidine, 0.05% digitonin, 0.01 % NP-40, 2% BSA, 1× protease inhibitors, and 10 mM MgCl_2_) and incubated for 1 h at 37 °C. The sample was mixed by pipetting after 30 min of the incubation to prevent sedimentation. The reaction was stopped by addition of 200 μl 1× diluted nuclei buffer (Chromium Next GEM Single Cell ATAC Library & Gel Bead Kit v1.1; 10x Genomics) supplemented with 2% BSA (1× DNB/BSA) and 12.5 mM EDTA. The nuclei were sedimented by centrifugation at 300*g* for 3 min and washed twice with 200 μl 1× DNB supplemented with 2% BSA. After the second wash and centrifugation, around 180 μl the 1× DNB/BSA supernatant was removed from the sample. The nuclei were resuspended in the remaining 20 μl 1× DNB/BSA, 2 μl were mixed with 8 μl trypan blue and counted manually using a counting chamber. Sixteen thousand nuclei were used to load the 10x chromium chip. Nano-CT without ATAC was performed in two biological replicates and, in parallel, nano-CT with ATAC was performed on the same biological sample as a technical replicate.

### Single-cell indexing and library prep

Single-cell indexing was performed using Chromium Next GEM Single Cell ATAC Library & Gel Bead Kit v1.1 (10x Genomics). Up to 8 μl nuclei suspension (filled up to 8 μl with 1× DNB/BSA) was mixed with 7 μl 10x ATAC buffer B, 56.5 μl barcoding reagent B, 1.5 μl reducing agent B and 1 μl barcoding enzyme. Seventy microliters of the master mixture was loaded on Chromium Next GEM Chip H together with 50 μl gel beads and 40 μl partitioning oil according to the manufacturer’s instructions followed by single-cell partitioning using the chromium controller and GEM incubation according to the manufacturer’s instructions (Chromium Next GEM Single Cell ATAC Reagent Kits v1.1 (Steps 2.0–2.5)).

Upon completion, the sample was recovered by post-GEM incubation cleanup with Dynabeads MyOne SILANE and SPRIselect beads according to Chromium Next GEM Single Cell ATAC Reagent Kits v1.1 (Steps 3.0–3.2)

After elution from SPRIselect beads, 40 μl sample was recovered and 2 μl were used to measure the concentration using Qubit dsDNA HS Assay kit (ThermoFisher). The rest of the sample was used for P7 tagmentation by mixing the following reaction: 38 μl template DNA, 50 μl 2× TD buffer (20 mM Tris pH 7.5, 10 mM MgCl2, and 20% dimethylformamide), 1 μl MeB-only loaded standard Tn5 (P7 Tn5; typically 0.5–1 μl P7 Tn5 in single-cell experiment; see above for details on loading conditions) per 10 ng template DNA and dH_2_O up to 100 μl and incubation for 30 min at 37 °C in a thermocycler. After that, the sample was purified with DNA Clean and concentrator-5 (Zymo) using 500 μl binding buffer and eluted in 40 μl Zymo elution buffer. The purified DNA was used as a template for the final PCR amplification according to Chromium Next GEM Single Cell ATAC Reagent Kits v1.1 (Step 4.1) (40 μl template, 50 μl AMP mix, 7.5 μl SI-PCR primer B and 2.5 μl individual Single index N Set A primer) and amplified using standard 10x scATAC-seq PCR amplification program (1. 98 °C 45 s; 2. 98 °C 20 s; 3. 67 °C 30 s; 4. 72 °C 20 s; 5. 72 °C 1 min; 6. 4 °C hold) and amplified for 11–15 cycles. The amplified product was purified with SPRIselect reagent (Beckman-Coulter) using 0.4× and 1.2× two-sided purification according to the Chromium Next GEM Single Cell ATAC Reagent Kits v1.1 (Step 4.2). The size distribution and concentration of the library was assessed using Qubit dsDNA HS Assay kit (ThermoFisher) and bioanalyzer high-sensitivity DNA kit. The sequencing library structure is shown in Supplementary data figure 2. The size distribution should be equal across 200-1000 bp range. Peak at cca 200bp or cca 1500bp means the sample has been overtagmented or undertagmented.

### Sequencing setup

The libraries were sequenced on Illumina Novaseq v1.5 using custom read1 (R1_seq GCGATCGAGGACGGCAGATGTGTATAAGAGACAG) and index2 (I2_seq CTGTCTCTTATACACATCTGCCGTCCTCGATCGC) primers (Supplementary Table 1) with custom read lengths 36-8-48-36 (R1-I1-I2-R2).

### Data analysis

#### Preprocessing

Raw fastq files were demultiplexed into individual fastq files for each modality using debarcode.py script (https://github.com/mardzix/bcd_nano_CUTnTag/blob/master/scripts/debarcode.py) with 1 allowed mismatch in barcode. Fastq files were then used as input into cellranger 1.2.0 pipeline, with modifications. Peaks were called using pseudo-bulk bam files outputted from cellranger (outs/possorted_bam.bam) using MACS2 with parameters ‘--keep-dup=1 --llocal 100000 --min-length 1000 --max-gap 1000 --broad-cutoff=0.1’. A custom script was used to identify single cells on the basis of the number of reads per cell and fraction of reads in peaks (counted using bedtools intersect) (Extended Data Fig. 3). A Seurat object was then created for each modality and replicate separately using R function FeatureMatrix and peaks called previously. Seurat objects were then merged per modality (ATAC, H3K27ac, and H3K27me3) and across the modalities.

#### Dimensionality reduction and clustering

All dimensionality reduction and clustering was performed using package Seurat v4.0.5 and Signac v1.4.0 under R v4.1.1. Dimensionality reduction was performed using LSI and UMAP. Clusters were annotated through integration with scRNA-seq data (mouse adolescent brain atlas)^34^ and manual annotation on the basis of marker genes. Bigwig files per cell cluster were constructing by filtering the possorted_bam.bam file using single-cell barcodes by custom script creating bam file per cluster and bamCoverage script from deeptools. Integration with scCUT&Tag and scRNA-seq was performed using CCA implemented in Seurat package v.4.0.5. Pseudo-time analysis was performed using Slingshot v2.2.0. Gene annotations from Ensembl release v79 were used in the analysis (EnsDb.Mmusculus.79).

#### Downstream analysis

Metagene plots were generated using deeptools package v.3.5.1 using computeMatrix and plotHeatmap scripts. Scores for individual peak regions were generated using deeptools multiBigwigSummary script. GO analysis was performed using R package clusterProfiler v.4.2.2. Correlation scatter plots were created using deepTools multiBigWigSummary script.

#### Chromatin dynamics analysis

Pseudo-time in the oligodendrocyte lineage was calculated using R package slingshot v.2.2.0^44^. Chromatin velocity was performed using Python package scvelo v.0.2.4 by directly using ATAC-seq and H3K27ac matrix gene-by-cell matrix into unspliced and spliced layers, respectively. scvelo analysis was performed using default parameters.

### Reporting summary

Further information on research design is available in the Nature Portfolio Reporting Summary linked to this article.

## Online content

Any methods, additional references, Nature Portfolio reporting summaries, source data, extended data, supplementary information, acknowledgements, peer review information; details of author contributions and competing interests; and statements of data and code availability are available at 10.1038/s41587-022-01535-4.

### Supplementary information

Supplementary Information Supplementary Sequences 1 and 2 and Supplementary Figs 1 and 2.

Reporting Summary

Supplementary Tables 1–4 Supplementary Table 1, list of oligonucleotide sequences. Supplementary Table 2, list of genes identified as the most important velocity driver genes by scvelo. Supplementary Table 3, summary of single-cell epigenome profiling technologies. Supplementary Table 4, detailed list of reagents.

## Data Availability

Nano-CUT&Tag data can be accessed and browsed through https://ki.se/en/mbb/oligointernode and the UCSC cell browser at https://mouse-epi-juv-brain.cells.ucsc.edu. Raw data was deposited in Gene Expression Omnibus under accession GSE198467. The following publicly available datasets were used in this study: GSE75330—scRNA-seq of oligodendrocyte lineage^41^; GSE163532—scCUT&Tag in the juvenile mouse brain^15^; SRP135960—scRNA-seq mouse juvenile brain atlas (http://mousebrain.org)^34^; scATAC-seq of adult mouse cortex (10x Genomics)^45^; and H3K27me3 chip-seq datasets from the ENCODE portal (https://www.encodeproject.org/) with the following identifiers ENCSR308TAV, ENCSR340ROY, and ENCSR070MOK^46^. The plasmids used for purification of nanobody-Tn5 fusions are available through Addgene (#183637 and #183638).

## Code availability

All code, scripts, analysis pipeline and instructions for reproducibility can be found on Github https://github.com/mardzix/bcd_nano_CUTnTag.
