# Supplementary material for: Multimodal chromatin profiling using nanobody-based single-cell CUT&Tag
Source: Nat Biotechnol. 2022 Dec 19;41(6):794–805. doi: 10.1038/s41587-022-01535-4 (PMC10264246; doi:10.1038/s41587-022-01535-4)
Supplement: Supplementary file 1 — Supplementary Sequences 1 and 2 and Supplementary Figs 1 and 2. [file 41587_2022_1535_MOESM1_ESM.pdf]

nature biotechnology

Article

https://doi.org/10.1038/s41587-022-01535-4

Multimodal chromatin profiling using nanobody-based single-cell CUT&Tag

In the format provided by the
authors and unedited

Supplementary sequence 1. Anti-mouse nanobody-Tn5 fusion protein sequence

>anti-Mouse-Tn5_protein_sequence
MHHHHHHSSGVDLGTENLYFQSGDYKDHDGDYKDHDIDYKDDDDKGGGSQVQLVESGGGWVQPGGSLR
LSCAASGFTFSDTAMMWVRQAPGKGREWVAAIDTGGGYTYYADSVKGRFTISRDNAKNTLYLQMNSLK
PEDTARYYCAKTYSGNYYSNYTVANYGTTGRGTLVTVSSGGGGSGGGGSGGGGSGGGGSHMITSALHR
AADWAKSVFSSAALGDPRRTARLVNVAAQLAKYSGKSITISSEGSKAMQEGAYRFIRNPNVSAEAIRK
AGAMQTVKLAQEFPELLAIEDTTSLSYRHQVAEELGKLGSIQDKSRGWWVHSVLLLEATTFRTVGLLH
QEWWMRPDDPADADEKESGKWLAAAATSRLRMGSMMSNVIAVCDREADIHAYLQDKLAHNERFVVRSK
HPRKDVESGLYLYDHLKNQPELGGYQISIPQKGVVDKRGKRKNRPARKASLSLRSGRITLKQGNITLN
AVLAEEINPPKGETPLKWLLLTSEPVESLAQALRVIDIYTHRWRIEEFHKAWKTGAGAERQRMEEPDN
LERMVSILSFVAVRLLQLRESFTPPQALRAQGLLKEAEHVESQSAETVLTPDECQLLGYLDKGKRKRK
EKAGSLQWAYMAIARLGGFMDSKRTGIASWGALWEGWEALQSKLDGFLAAKDLMAQGIKI

6x HIS tag
TEV cleavage site
3xFLAG tag
Anti-mouse nanobody
4x GGGGS linker
Tn5 transposase

>anti-Mouse-Tn5_DNA_Sequence
ATGCACCACCACCATCACCATAGTAGCGGAGTCGACTTGGGCACCGAGAATCTGTACTTTCAAAG
TGGCGATTACAAGGACCACGACGGCGACTACAAGGACCATGACATCGACTATAAGGATGATGATG
ACAAGGGCGGTGGTTCACAAGTCCAATTGGTTGAGAGTGGCGGTGGTTGGGTTCAACCGGGTGGT
TCATTGCGTCTCAGTTGTGCCGCGAGCGGCTTTACATTTTCAGACACCGCCATGATGTGGGTACG
TCAAGCTCCGGGCAAGGGGCGTGAGTGGGTGGCTGCCATCGACACTGGTGGCGGCTATACTTACT
ATGCGGACTCCGTCAAAGGCCGTTTTACGATCTCCCGTGACAATGCGAAGAATACGTTATATCTC
CAAATGAATTCGCTCAAGCCTGAAGACACCGCGCGTTATTATTGTGCGAAGACGTATTCAGGGAA
TTACTACAGTAATTACACGGTTGCTAACTACGGGACGACCGGTCGTGGAACCTTGGTTACGGTTT
CAAGCGGTGGTGGTGGAAGTGGTGGTGGTGGGTCGGGTGGTGGTGGTTCTGGCGGTGGCGGAAGC
CACATGATTACGTCAGCGTTGCACCGTGCTGCGGACTGGGCGAAGAGCGTGTTCTCAAGCGCAGC
ACTTGGTGATCCTCGTCGTACGGCCCGCCTGGTGAATGTTGCGGCCCAACTGGCCAAATACAGTG
GTAAATCAATTACGATTAGTTCGGAAGGCAGCAAGGCAATGCAGGAAGGTGCATACCGCTTCATC
CGTAATCCCAACGTTAGTGCGGAAGCCATTCGCAAGGCAGGCGCGATGCAGACAGTGAAATTAGC
ACAGGAATTTCCGGAATTACTTGCGATTGAGGATACAACGAGCTTATCGTATCGTCATCAAGTCG
CGGAAGAACTGGGCAAATTAGGGTCAATCCAGGATAAAAGCCGTGGTTGGTGGGTGCATTCAGTT
CTGCTGCTGGAGGCGACCACTTTTCGCACGGTGGGCCTGTTACATCAAGAGTGGTGGATGCGCCC
GGATGATCCCGCGGACGCGGATGAAAAGGAGAGCGGCAAATGGCTCGCGGCCGCGGCCACCTCCC
GCCTGCGTATGGGCAGCATGATGTCGAACGTGATCGCCGTGTGCGATCGCGAAGCGGATATTCAC
GCGTACCTGCAGGACAAACTGGCTCATAATGAACGGTTTGTGGTGCGCTCTAAACATCCGCGTAA
AGACGTTGAATCCGGCTTATACCTCTACGACCATCTGAAGAACCAACCAGAGCTTGGTGGGTACC
AGATTTCTATTCCGCAGAAGGGTGTAGTGGATAAACGTGGTAAACGTAAAAATCGCCCAGCCCGT
AAAGCGAGCTTGAGCTTGCGTTCGGGTCGTATCACGTTAAAACAAGGTAACATCACCCTCAATGC
AGTGCTGGCTGAGGAAATTAATCCACCGAAAGGTGAGACCCCGCTGAAATGGCTGCTGCTGACTA
GCGAACCGGTGGAATCCCTGGCGCAGGCGCTTCGTGTGATTGATATTTACACCCATCGTTGGCGT
ATCGAGGAATTTCACAAGGCGTGGAAAACAGGCGCAGGCGCCGAACGCCAGCGTATGGAAGAGCC
AGATAACCTGGAGCGTATGGTATCGATCCTGTCGTTCGTGGCTGTGCGTCTTCTGCAGCTGCGCG
AGTCCTTCACTCCGCCGCAGGCCTTACGGGCTCAGGGCCTTCTGAAGGAAGCCGAGCACGTTGAA
TCGCAAAGCGCCGAAACGGTGCTGACACCGGACGAGTGCCAGTTACTGGGTTATTTAGACAAGGG
CAAGCGGAAACGCAAAGAAAAAGCCGGCTCCCTTCAGTGGGCCTATATGGCAATCGCACGTCTCG
GCGGCTTTATGGATTCGAAACGCACCGGCATTGCAAGCTGGGGCGCTCTGTGGGAGGGTTGGGAA
GCGCTGCAGAGTAAGCTGGATGGTTTTTTGGCTGCTAAAGACTTGATGGCTCAGGGAATCAAAAT
CTAA

Supplementary sequence 2. Anti-mouse nanobody-Tn5 fusion protein sequence

>anti-Rabbit-Tn5_protein_sequence
MHHHHHHSSGVDLGTENLYFQSGDYKDHDGDYKDHDIDYKDDDDKGGGSQVQLVESGGGLVQAGDSLR
LSCVASGRSLDGATMRWYRQAPGKEREFVAGIFWDEIGTEYADTAKGRFTISRDNAKNTIYLQMTNLR
SEDTAMYYCNGLVFGGEYWGQGTQVTVSSGGGGSGGGGSGGGGSGGGGSHMITSALHRAADWAKSVFS
SAALGDPRRTARLVNVAAQLAKYSGKSITISSEGSKAMQEGAYRFIRNPNVSAEAIRKAGAMQTVKLA
QEFPELLAIEDTTSLSYRHQVAEELGKLGSIQDKSRGWWVHSVLLLEATTFRTVGLLHQEWWMRPDDP
ADADEKESGKWLAAAATSRLRMGSMMSNVIAVCDREADIHAYLQDKLAHNERFVVRSKHPRKDVESGL
YLYDHLKNQPELGGYQISIPQKGVVDKRGKRKNRPARKASLSLRSGRITLKQGNITLNAVLAEEINPP
KGETPLKWLLLTSEPVESLAQALRVIDIYTHRWRIEEFHKAWKTGAGAERQRMEEPDNLERMVSILSF
VAVRLLQLRESFTPPQALRAQGLLKEAEHVESQSAETVLTPDECQLLGYLDKGKRKRKEKAGSLQWAY
MAIARLGGFMDSKRTGIASWGALWEGWEALQSKLDGFLAAKDLMAQGIKI

6x HIS tag
TEV cleavage site
3xFLAG tag
Anti-rabbit nanobody
4x GGGGS linker
Tn5 transposase

>anti-Rabbit-Tn5_DNA_Sequence
ATGCATCACCACCACCACCATTCTTCTGGCGTCGACTTGGGTACGGAGAATCTGTATTTTCAATC
GGGAGACTACAAGGACCACGATGGTGACTATAAGGATCACGATATCGACTATAAAGACGACGACG
ACAAGGGTGGCGGTTCCCAAGTACAATTGGTCGAGTCAGGCGGTGGTCTTGTACAAGCAGGTGAC
AGCTTACGCCTGAGTTGTGTTGCGAGTGGACGCAGCCTTGACGGAGCCACGATGCGCTGGTACCG
CCAAGCGCCTGGGAAGGAGCGTGAGTTTGTGGCGGGTATCTTCTGGGACGAGATTGGCACTGAGT
ACGCGGACACCGCCAAGGGTCGCTTTACGATTTCTCGCGACAATGCCAAGAACACCATTTATTTG
CAAATGACAAATCTGCGCTCTGAGGACACTGCTATGTATTATTGTAATGGACTCGTATTTGGCGG
AGAGTATTGGGGCCAGGGCACCCAAGTAACTGTGAGCTCTGGCGGCGGTGGCTCAGGTGGTGGTG
GTTCCGGTGGCGGCGGTTCTGGTGGTGGTGGAAGTCACATGATCACGTCTGCCCTGCATCGTGCC
GCGGACTGGGCCAAGAGCGTCTTCAGCTCCGCTGCGTTGGGCGATCCCCGCCGTACGGCGCGCCT
GGTGAACGTTGCCGCACAGCTTGCGAAATATAGCGGTAAAAGTATCACGATCAGCAGTGAGGGTA
GCAAAGCCATGCAAGAGGGTGCGTATCGTTTCATCCGCAATCCGAATGTATCGGCTGAAGCAATT
CGTAAAGCCGGGGCTATGCAGACCGTGAAACTTGCCCAAGAATTTCCTGAACTCCTGGCAATCGA
AGATACCACCTCCCTGTCGTACCGGCATCAGGTTGCGGAAGAACTGGGGAAACTGGGCAGTATCC
AGGACAAATCACGGGGATGGTGGGTCCACTCTGTTCTGTTACTTGAAGCAACAACCTTTCGCACG
GTGGGCCTTCTCCATCAGGAATGGTGGATGCGCCCAGATGACCCCGCCGATGCGGATGAAAAAGA
AAGCGGGAAGTGGCTTGCTGCGGCCGCAACGTCCCGGTTACGTATGGGCAGCATGATGAGCAACG
TGATTGCAGTGTGCGATCGTGAAGCCGACATTCACGCTTATTTGCAGGACAAATTAGCGCACAAT
GAACGCTTCGTAGTCCGTTCGAAACATCCGCGTAAAGACGTTGAATCTGGCCTGTACCTGTATGA
TCACCTGAAAAACCAGCCCGAGCTGGGCGGTTATCAGATCAGCATTCCGCAGAAAGGAGTGGTTG
ATAAGCGCGGCAAACGCAAAAACCGTCCGGCGCGTAAAGCTAGCCTTAGCTTACGTTCTGGGCGC
ATCACGCTTAAACAGGGTAACATTACGCTCAATGCAGTCTTAGCAGAAGAAATCAACCCTCCGAA
AGGTGAAACGCCACTGAAATGGCTGCTGCTCACCAGCGAACCAGTGGAGAGTTTGGCACAAGCTC
TGCGCGTAATTGATATCTACACTCATCGCTGGCGGATTGAAGAATTTCACAAAGCGTGGAAAACC
GGTGCCGGCGCAGAACGTCAGCGTATGGAGGAACCGGATAATCTGGAGCGCATGGTGTCTATTCT
GTCATTCGTTGCGGTTCGCTTGCTGCAGCTGCGCGAATCGTTTACGCCTCCGCAGGCGCTCCGTG
CACAGGGTCTGTTAAAAGAGGCGGAACATGTGGAAAGTCAGTCGGCTGAGACTGTGCTGACGCCA
GACGAATGTCAGTTGTTGGGGTATCTGGATAAAGGCAAACGCAAACGGAAAGAGAAAGCCGGCTC
CCTCCAATGGGCGTACATGGCAATCGCACGTCTGGGCGGCTTCATGGATAGCAAACGTACCGGTA
TTGCTAGCTGGGGTGCCCTGTGGGAAGGATGGGAAGCCCTGCAGTCGAAACTGGATGGCTTCCTG
GCCGCGAAAGATCTGATGGCGCAAGGTATCAAGATTTGA

Supplementary figure 1

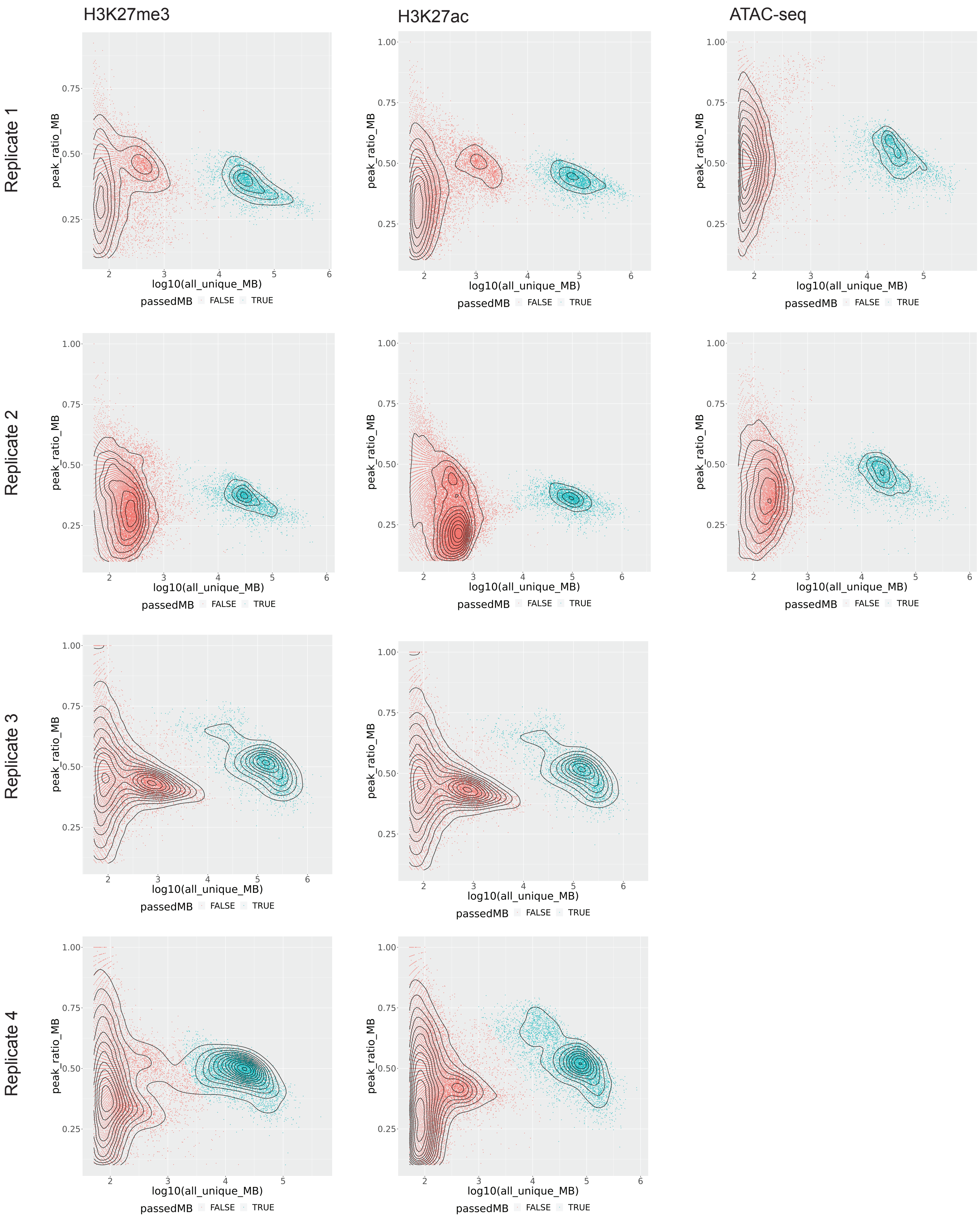

Supplementary data figure 1. a. Scatter plot showing number of reads per cell (x-axis) versus fraction of reads in peak regions (y-axis). Gaussian mixed model clustering was used to identify clusters of cells and cluster with the highest median of number of reads was selected as valid cells. Valid cells are labeled with cyan, while cells that do not pass QC are red. Contour lines depict density of points withing regions.

Supplementary figure 2

a

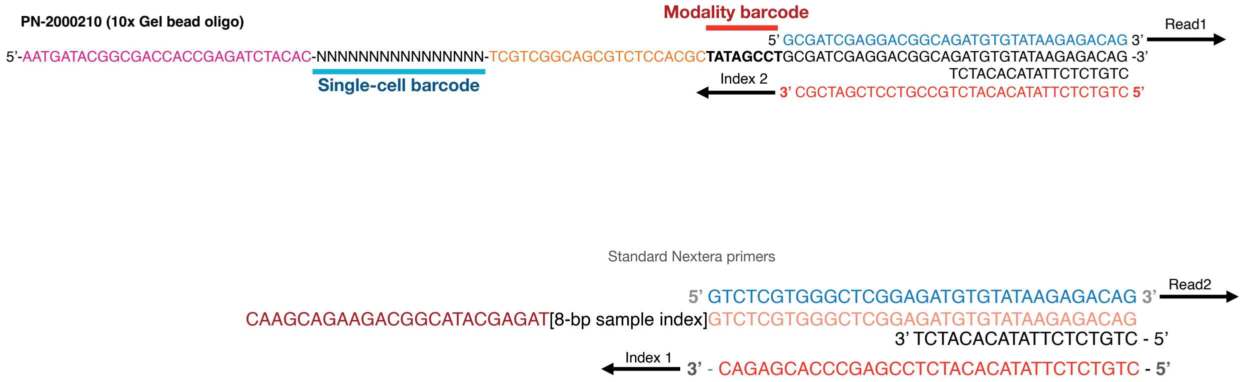

R1	36	Custom
R2	36	Standard
I1	8	Standard
I2	48	Custom

130 cycles max

Custom R1 and I2 primer

Custom_primer_R1: GCGATCGAGGACGGCAGATGTGTATAAGAGACAG
Custom_primer_I2: CTGTCTCTTATACACATCTGCCGTCCTCGATCGC

Supplementary data figure 2

a. Structure of the nano-CT library with highlighted barcode regions.
