# Supplementary material for: Multimodal chromatin profiling using nanobody-based single-cell CUT&Tag
Source: Nat Biotechnol. 2022 Dec 19;41(6):794–805. doi: 10.1038/s41587-022-01535-4 (PMC10264246; doi:10.1038/s41587-022-01535-4)
Supplement: Supplementary file 2 — Reporting Summary [file 41587_2022_1535_MOESM2_ESM.pdf]

Corresponding author(s): Marek Bartosovic, Gonçalo Castelo-Branco

Last updated by author(s): Sep 26, 2022

Reporting Summary

Nature Research wishes to improve the reproducibility of the work that we publish. This form provides structure for consistency and transparency in reporting. For further information on Nature Research policies, see our Editorial Policies and the Editorial Policy Checklist.

Statistics

For all statistical analyses, confirm that the following items are present in the figure legend, table legend, main text, or Methods section.

n/a	Confirmed	
☐	☒	The exact sample size (*n*) for each experimental group/condition, given as a discrete number and unit of measurement
☐	☒	A statement on whether measurements were taken from distinct samples or whether the same sample was measured repeatedly
☐	☒	The statistical test(s) used AND whether they are one- or two-sided<br>*Only common tests should be described solely by name; describe more complex techniques in the Methods section.*
☒	☐	A description of all covariates tested
☒	☐	A description of any assumptions or corrections, such as tests of normality and adjustment for multiple comparisons
☐	☒	A full description of the statistical parameters including central tendency (e.g. means) or other basic estimates (e.g. regression coefficient) AND variation (e.g. standard deviation) or associated estimates of uncertainty (e.g. confidence intervals)
☐	☒	For null hypothesis testing, the test statistic (e.g. F, t, r) with confidence intervals, effect sizes, degrees of freedom and P value noted<br>*Give P values as exact values whenever suitable.*
☒	☐	For Bayesian analysis, information on the choice of priors and Markov chain Monte Carlo settings
☒	☐	For hierarchical and complex designs, identification of the appropriate level for tests and full reporting of outcomes
☒	☐	Estimates of effect sizes (e.g. Cohen's *d*, Pearson's *r*), indicating how they were calculated

Our web collection on statistics for biologists contains articles on many of the points above.

Software and code

Policy information about availability of computer code

Data collection	Samples were sequenced on NovaSeq6000 (NovaSeq Control Software 1.7.5/RTA v3.4.4) with a 36nt(Read1)-8nt(Index1)-48nt(Index2)-36nt(Read2) setup using 'NovaSeqStandard' workflow in 'SP' mode flowcell. The Bcl to FastQ conversion was performed using bcl2fastq_v2.20.0.422 from the CASAVA software suite. The quality scale used is Sanger / phred33 / Illumina 1.8+
Data analysis	Raw fastq files were demultiplexed into individual fastq files for each modality using debarcode.py script (https://github.com/mardzix/bcd_nano_CUTnTag/blob/e8befad7d52ad60fc984a0c06b7b8496c1835638/scripts/debarcode.py) with 1 allowed mismatch in barcode. Fastq files were then used as input into cellranger 1.2.0 pipeline, with modifications. Data was analyzed using combination of published tools and custom scripts. All code needed to generate the figures is published at https://github.com/mardzix/bcd_nano_CUTnTag.Data was analyzed using combination of published tools and custom scripts. All code needed to generate the figures is published at https://github.com/mardzix/bcd_nano_CUTnTag.

For manuscripts utilizing custom algorithms or software that are central to the research but not yet described in published literature, software must be made available to editors and reviewers. We strongly encourage code deposition in a community repository (e.g. GitHub). See the Nature Research guidelines for submitting code & software for further information.

Data

Policy information about availability of data

All manuscripts must include a data availability statement. This statement should provide the following information, where applicable:

- Accession codes, unique identifiers, or web links for publicly available datasets
- A list of figures that have associated raw data
- A description of any restrictions on data availability

Raw data was deposited in GEO under accession GSE19846746. The following publicly available datasets were used in this study GSE75330 - scRNA-seq of oligodendrocyte lineage, GSE163532 - scCUT&Tag in the juvenile mouse brain, SRP135960 - scRNA-seq, Mouse juvenile brain atlas (http://mousebrain.org), scATAC-seq of adult mouse cortex (10x Genomics), H3K27me3 chip-seq datasets from the ENCODE portal (https://www.encodeproject.org/) with the following identifiers: ENCSR308TAV, ENCSR340ROY and ENCSR070MOK48.

Field-specific reporting

Please select the one below that is the best fit for your research. If you are not sure, read the appropriate sections before making your selection.

☒ Life sciences ☐ Behavioural & social sciences ☐ Ecological, evolutionary & environmental sciences

For a reference copy of the document with all sections, see nature.com/documents/nr-reporting-summary-flat.pdf

Life sciences study design

All studies must disclose on these points even when the disclosure is negative.

Sample size	No sample size calculation was performed to pre-determine sample sizes. The sample sizes were in the same range of other studies in the field.<br>The unimodal nano-CT was performed in 1 biological replicate. The multimodal nano-CT was performed in 2 biological replicates.
Data exclusions	For Nano-CT individual thresholds were set for number of reads, number of unique reads, percentage of reads in peaks, and number of reads in blacklist regions per cell.
Replication	One biological replicate of P19 brain for the original H3K27me3 nano-CT (Figure 1). Two biological replicates of P19 brain were analysed for Nano-CT for H3K27ac and H3K27me3, and two biological replicates of P19 brain were analysed for Nano-CT for H3K27ac and H3K27me3, coupled with ATAC.<br><br>We assessed the replication by examination of intermingling of cells originating from different experiments in clusters and in low-dimensional UMAP space.
Randomization	Randomization was not applied. Given that brain samples were collected at one unique time point, co-variate analysis is not relevant to the study. Animals from multiple litters over a course of approximately 6 months were used for the experiments.
Blinding	Given that brain samples were collected at one unique time point, blinding was not performed in this study. The clustering of the Nano-CT data was unsupervised.

Reporting for specific materials, systems and methods

We require information from authors about some types of materials, experimental systems and methods used in many studies. Here, indicate whether each material, system or method listed is relevant to your study. If you are not sure if a list item applies to your research, read the appropriate section before selecting a response.

Materials & experimental systems

n/a	Involved in the study	
☐	☒	Antibodies
☒	☐	Eukaryotic cell lines
☒	☐	Palaeontology and archaeology
☐	☒	Animals and other organisms
☒	☐	Human research participants
☒	☐	Clinical data
☒	☐	Dual use research of concern

Methods

n/a	Involved in the study	
☒	☐	ChIP-seq
☒	☐	Flow cytometry
☒	☐	MRI-based neuroimaging

Antibodies

Antibodies used

The following antibodies were used in the multimodal nano-CT experiments: mouse anti-H3K27me3 (Abcam, Ab6002), rabbit anti-H3K27ac (Abcam Ab177178). Anti-H3K27me3 (Cell Signaling, 9733T) was used in single modality nano-CT experiment.

Validation

All antibodies used in this study have been validated and tested by the provider company and/or have been cited by other authors, references are available on the web page of the provider company.

Ab6002
Specificity
This antibody is specific for histone H3 tri-methylated at K27. The antibody is blocked in Western blot by tri methyl K27 peptide and slightly by di methyl K27 peptide (there is <12% cross reactivity with di methyl K27 as determined by ELISA). It is not blocked by mono methyl K4, di methyl K4, tri methyl K4, mono methyl K9, di methyl K9, tri methyl K9, mono methyl K27 or unmodified K27 peptides (see the peptide blocking assay blot below).

References:
Zhang H et al. m6A methyltransferase METTL3-induced lncRNA SNHG17 promotes lung adenocarcinoma gefitinib resistance by epigenetically repressing LATS2 expression. Cell Death Dis 13:657 (2022). ChIP ; Human .PubMed: 35902569

Liu L et al. KDM6A-ARHGDIB axis blocks metastasis of bladder cancer by inhibiting Rac1. Mol Cancer 20:77 (2021).PubMed: 34006303

An Q et al. Calcitonin gene-related peptide regulates spinal microglial activation through the histone H3 lysine 27 trimethylation via enhancer of zeste homolog-2 in rats with neuropathic pain. J Neuroinflammation 18:117 (2021).PubMed: 34020664

Davenport KM et al. Characterizing Genetic Regulatory Elements in Ovine Tissues. Front Genet 12:628849 (2021).PubMed: 34093640

Huang Y et al. Tanshinone I, a new EZH2 inhibitor restricts normal and malignant hematopoiesis through upregulation of MMP9 and ABCG2. Theranostics 11:6891-6904 (2021).PubMed: 34093860View all Publications for this product

9733T
Specificity / Sensitivity
Tri-Methyl-Histone H3 (Lys27) (C36B11) Rabbit mAb detects endogenous levels of histone H3 only when tri-methylated on Lys27. The antibody does not cross-react with non-methylated, mono-methylated or di-methylated Lys27. In addition, the antibody does not cross-react with mono-methylated, di-methylated or tri-methylated histone H3 at Lys4, Lys9, Lys36 or Histone H4 at Lys20.

References:
Wang X, Rosikiewicz W, Sedkov Y, Mondal B, Martinez T, Kallappagoudar S, Tvardovskiy A, Bajpai R, Xu B, Pruett-Miller SM, Schneider R, Herz HM. The MLL3/4 complexes and MiDAC co-regulate H4K20ac to control a specific gene expression program. Life Sci Alliance. 2022 Jul 12;5(11):e202201572. doi: 10.26508/lsa.202201572. PMID: 35820704; PMCID: PMC9275676.

Thompson JJ, Lee DJ, Mitra A, Frail S, Dale RK, Rocha PP. Extensive co-binding and rapid redistribution of NANOG and GATA6 during emergence of divergent lineages. Nat Commun. 2022 Jul 23;13(1):4257. doi: 10.1038/s41467-022-31938-5. PMID: 35871075; PMCID: PMC9308780.

Jiang M, Qi F, Zhang K, Zhang X, Ma J, Xia S, Chen L, Yu Z, Chen J, Chen D. MARCKSL1-2 reverses docetaxel-resistance of lung adenocarcinoma cells by recruiting SUZ12 to suppress HDAC1 and elevate miR-200b. Mol Cancer. 2022 Jul 21;21(1):150. doi: 10.1186/s12943-022-01605-w. PMID: 35864549; PMCID: PMC9306054.

Rhodes CT, Thompson JJ, Mitra A, Asokumar D, Lee DR, Lee DJ, Zhang Y, Jason E, Dale RK, Rocha PP, Petros TJ. An epigenome atlas of neural progenitors within the embryonic mouse forebrain. Nat Commun. 2022 Jul 20;13(1):4196. doi: 10.1038/s41467-022-31793-4. PMID: 35858915; PMCID: PMC9300614.

Barnes RP, de Rosa M, Thosar SA, Detwiler AC, Roginskaya V, Van Houten B, Bruchez MP, Stewart-Ornstein J, Opresko PL. Telomeric 8-oxo-guanine drives rapid premature senescence in the absence of telomere shortening. Nat Struct Mol Biol. 2022 Jul;29(7):639-652. doi: 10.1038/s41594-022-00790-y. Epub 2022 Jun 30. PMID: 35773409; PMCID: PMC9287163.

Ab177178
Specificity
ab177178 binds K27ac alone and also when S28 is phosphorylated

References:
Jin Q et al. lncRNA MIR22HG-Derived miR-22-5p Enhances the Radiosensitivity of Hepatocellular Carcinoma by Increasing Histone Acetylation Through the Inhibition of HDAC2 Activity. Front Oncol 11:572585 (2021).PubMed: 33718133
Liu Y et al. Long noncoding RNA LINC00518 induces radioresistance by regulating glycolysis through an miR-33a-3p/HIF-1a negative feedback loop in melanoma. Cell Death Dis 12:245 (2021).PubMed: 33664256
Azambuja AP & Simoes-Costa M A regulatory sub-circuit downstream of Wnt signaling controls developmental transitions in neural crest formation. PLoS Genet 17:e1009296 (2021).PubMed: 33465092
Cheng M et al. Transcription Factor ELF1 Activates MEIS1 Transcription and Then Regulates the GFI1/FBW7 Axis to Promote the Development of Glioma. Mol Ther Nucleic Acids 23:418-430 (2021).PubMed: 33473327
Liu X et al. IL-9-triggered lncRNA Gm13568 regulates Notch1 in astrocytes through interaction with CBP/P300: contribute to the pathogenesis of experimental autoimmune encephalomyelitis. J Neuroinflammation 18:108 (2021).PubMed: 33971906
Ye X et al. Oncostatin M Maintains Naïve Pluripotency of mESCs by Tetraploid Embryo Complementation (TEC) Assay. Front Cell Dev

Biol 9:675411 (2021).PubMed: 34124061
Takayama KI et al. Subtype-specific collaborative transcription factor networks are promoted by OCT4 in the progression of prostate cancer. Nat Commun 12:3766 (2021).PubMed: 34145268
Ehteda A et al. Dual targeting of the epigenome via FACT complex and histone deacetylase is a potent treatment strategy for DIPG. Cell Rep 35:108994 (2021).PubMed: 33852836
Sarode GV et al. Wilson Disease: Intersecting DNA Methylation and Histone Acetylation Regulation of Gene Expression in a Mouse Model of Hepatic Copper Accumulation. Cell Mol Gastroenterol Hepatol 12:1457-1477 (2021).PubMed: 34098115
Zhang Y et al. Nuclear condensates of p300 formed though the structured catalytic core can act as a storage pool of p300 with reduced HAT activity. Nat Commun 12:4618 (2021).

Animals and other organisms

Policy information about studies involving animals; ARRIVE guidelines recommended for reporting animal research

Laboratory animals

The mouse line used in this study was generated by crossing Sox10:Cre animals (The Jackson Laboratory mouse strain 025807) on a C57BL/6j genetic background with RCE:loxP-STOP-loxP (EGFP) animals (The Jackson Laboratory mouse strain 32037-JAX) on a C57BL/6xCD1 mixed genetic background. Animals of both sexes were sacrificed at P19.

All animals were free from mouse viral pathogens, ectoparasites and endoparasites and mouse bacteria pathogens. Mice were kept with the following light/dark cycle: dawn 6:00-7:00, daylight 7:00-18:00, dusk 18:00-19:00, night 19:00-6:00 and housed to a maximum number of 5 per cage in individually ventilated cages (IVC sealsafe GM500, tecniplast). General housing parameters such as relative humidity, temperature, and ventilation follow the European convention for the protection of vertebrate animals used for experimental and other scientific purposes treaty ETS 123, Strasbourg 18.03.1996/01.01.1991. Briefly, consistent relative air humidity of 55%±10, 22 °C and the air quality is controlled with the use of stand-alone air handling units supplemented with HEPA filtrated air. Monitoring of husbandry parameters is done using ScanClime® (Scanbur) units. Cages contained hardwood bedding (TAPVEI, Estonia), nesting material, shredded paper, gnawing sticks and card box shelter (Scanbur). The mice received regular chow diet (either R70 diet or R34, Lantmännen Lantbruk, Sweden). Water was provided by using a water bottle, which was changed weekly. Cages were changed every other week. All cage changes were done in a laminar air-flow cabinet.�Facility personnel wore dedicated scrubs, socks and shoes. Respiratory masks were used when working outside of the laminar air-flow cabinet.

Wild animals

No wild animals were used in this study

Field-collected samples

No field-collected samples were used in this study

Ethics oversight

All experimental procedures on animals were performed following the European directive 2010/63/EU, local Swedish directive L150/SJVFS/2019:9, Saknr L150 and Karolinska Institutet complementary guidelines for procurement and use of laboratory animals, Dnr. 1937/03-640. The procedures described were approved by the local committee for ethical experiments on laboratory animals in Sweden (Stockholms Norra Djurförsöksetiska nämnd), lic. nr. 144/16 and 1995_2019.

Note that full information on the approval of the study protocol must also be provided in the manuscript.
